# Immunologic and placental responses to the VSV-EBOV vaccine in a pregnant mouse model

**DOI:** 10.1172/jci.insight.201907

**Published:** 2026-10-22

**Authors:** Aaron P. Lewkowicz, Yiru Wang, Mirian I. Mendoza, Kaliroi Engel, Logan Kelley-Baker, Iara Rattner, Amber Lopez, Ian L. McWilliams, Derek D.C. Ireland, Mohanraj Manangeeswaran, Ha-Na Lee, Daniela Verthelyi, Sandra M. Blois

**Affiliations:** 1 Office of Pharmaceutical Quality Research–Division IV, Office of Pharmaceutical Quality, Center for Drug Evaluation and Research, Food and Drug Administration, Silver Spring, Maryland, USA.; 2 Department of Obstetrics and Fetal Medicine, University Medical Center Hamburg-Eppendorf (UKE), Germany.; 3 Division of Veterinary Services, Center for Biologics Evaluation and Research, Food and Drug Administration, Silver Spring, Maryland, USA.; 4 Glyco-HAM, a cooperation of Universität Hamburg (UHH), Technology Platform Mass Spectrometry, and University Medical Center Hamburg-Eppendorf (UKE), Hamburg, Germany.

**Keywords:** Infectious disease, Reproductive biology, Reproductive biochemistry, Vaccines

## Abstract

Ebola virus disease is a severe viral hemorrhagic illness with high maternal and perinatal mortality. Although the replication-competent vesicular stomatitis virus–based Ebola vaccine (VSV-EBOV) has been deployed successfully in outbreak settings, pregnant women were excluded from clinical trials, leaving critical safety gaps. To address this, we developed a murine model to assess pregnancy outcomes following VSV-EBOV exposure at different gestational stages. Pregnant C57BL/6 mice vaccinated at embryonic day 7 (E7), E10, or E15 mounted robust anti–Ebola virus (anti-EBOV) antibody responses comparable to those of non-pregnant controls and efficiently cleared the vaccine virus without evidence of vertical transmission. However, peri-implantation vaccination modestly increased fetal resorption and early postnatal mortality, accompanied by placental pathology characterized by vascular remodeling defects and inflammation. Later gestational vaccination had minimal impact on survival or offspring development. Mechanistic studies revealed that these adverse effects were mediated by type I interferon signaling rather than direct viral infection, as IFN-β–deficient mice exhibited normal pregnancy outcomes and blocking interferon receptor activity ameliorated placental pathology despite higher viral loads. To our knowledge, these findings represent the first comprehensive preclinical safety evaluation of VSV-EBOV vaccination during pregnancy and identify a critical window of vulnerability early in gestation. More broadly, this work underscores the central role of interferons in shaping placental immune responses and provides an experimental framework to guide evaluation of live-virus vaccines in pregnancy.

## Abstract 2

First mouse safety study of Ebola vaccine in pregnancy finds it largely safe, but early vaccination can raise fetal loss via interferon signaling

## Introduction

Ebola virus (EBOV) is a member of the Filoviridae family, recognized as one of the most virulent pathogens and the causative agent of Ebola virus disease (EVD), a severe and often fatal illness with mortality rates that can reach up to 90% (1, 2). The clinical presentation of EVD is diverse, ranging from initial nonspecific symptoms such as fever, malaise, and diarrhea to more severe manifestations including dehydration, neurological complications, systemic hemorrhage, multi-organ failure, and shock, often culminating in death (3). Since the virus was first identified in 1976 near the Ebola River in the Democratic Republic of the Congo, it has been responsible for numerous outbreaks and epidemics, claiming tens of thousands of lives (4, 5). With recurring outbreaks still posing significant global health threats, the need for effective and safe vaccination remains urgent (6). Although over 70 clinical trials have been conducted to develop different vaccines against EBOV, to date, only ERVEBO has received US Food and Drug Administration approval (6). ERVEBO is a live attenuated vaccine that uses a replication-competent pseudotyped vesicular stomatitis virus (VSV) platform in which endogenous glycoprotein (GP) is replaced with Zaire EBOV-GP (rVSVΔG-EBOV-GP; also known as VSV-EBOV). Multiple clinical trials have demonstrated that this vaccine is well tolerated without serious adverse events; however, the efficacy and safety of VSV-EBOV during pregnancy have not been established (7). A study of 84 women inadvertently vaccinated in early pregnancy or who became pregnant less than 60 days after vaccination showed a slight increase in pregnancy loss (45% vs. 33% in vaccinated vs. unvaccinated women), but the results were inconclusive (8).

Pregnancy induces progressive hormonal shifts that cause immunologic adaptations, potentially increasing susceptibility to infections or impairing virus clearance; therefore, pregnant women are at a higher risk for several infectious diseases, including influenza virus, hepatitis E virus, and herpes simplex virus (9). Physiological changes associated with pregnancy, including cardiovascular, respiratory, endocrine, and metabolic adaptations, may also contribute to disease severity (10). Although there is no direct evidence suggesting increased susceptibility to EBOV during pregnancy, several studies suggest that pregnant women with EVD face a significantly higher risk of severe complications, including fetal loss and pregnancy-related hemorrhage (11). Moreover, the pathophysiology of EVD in pregnancy is complicated by the ability of EBOV to persist in the tolerogenic milieu of the placenta, potentially exacerbating the risk to both mother and fetus (12). Vaccination is a crucial strategy to protect pregnant women and their unborn children from the severe consequences of EVD. However, because of concerns about potential risks to maternal and fetal health, pregnant women were excluded from the pivotal clinical trials of various vaccines, so their safety and efficacy in this population remain unclear (13).

In previous studies, we used VSV-EBOV to develop a biosafety level 2–compatible symptomatic murine model of Ebola to investigate viral tropism mediated by the Ebola Zaire glycoprotein (EBOV-GP) and test therapeutics targeting this glycoprotein. Subcutaneous inoculation of neonatal C57BL/6 mice with VSV-EBOV led to weight loss, meningoencephalitis, neurodegeneration, retinitis, and death 7–12 days after administration (14–16). The disease was EBOV-GP dependent, as mice challenged with VSV bearing the glycoprotein from EBOV Reston did not develop a fatal infection (14). Based on these findings, we reasoned that the VSV-EBOV vaccine could be used to study host-pathogen interactions and assess the effect of therapeutics targeting EBOV-GP in clearing virus from immune-privileged sites (15, 16). Unlike neonatal mice, adult wild-type C57BL/6 mice are resistant to VSV-EBOV challenge as evidenced by rapid virus clearance and an absence of disease symptoms (14). Since pregnancy influences the immune status of the dam, here we investigated whether VSV-EBOV would induce protective antibody titers in dams and examined the potential impact of administering VSV-EBOV challenge to pregnant C57BL/6 female mice and their offspring.

## Results

### Vaccination of pregnant C57BL/6 mice with VSV-EBOV.

To evaluate the suitability of pregnant C57BL/6 mice as a model for studying the Ebola vaccine, we first assessed whether pregnant dams would mount an appropriate antibody response to VSV-EBOV. Pregnant dams were inoculated with 10^5^ TCID_50_ (50% tissue culture infectious dose) of VSV-EBOV on embryonic day 7 (E7), E10, and E15, representing peri-implantation, mid-pregnancy, and late pregnancy, respectively (Figure 1A). The selected dosage was based on a 250-fold reduction of viral load used in human trials to proportionally adjust for the smaller size of mice (17, 18). At 21 and 42 days post-challenge (dpc), the antibody titers against VSV-EBOV were comparable in pregnant mice to those observed in non-pregnant female mice (Figure 1B). Importantly, weight gain in vaccinated dams was similar to that in unvaccinated dams (Figure 1C), and there were no significant differences in litter size between control and vaccinated groups (Figure 1D).

Assessment of the offspring showed a statistically significant reduction in newborn birth weight across all inoculated groups (Figure 1E). Vaccination on E7 resulted in a marginal increase in pregnancy losses (i.e., no offspring despite confirmed pregnancy), suggesting a potential impact of VSV-EBOV exposure during the peri-implantation period (Figure 1F and Supplemental Table 1; supplemental material available online with this article; https://doi.org/10.1172/jci.insight.201907DS1). In addition, among dams challenged at E7, there was an increase in the number of litters that had one or more pups that died during the first week of life (Figure 1G and Supplemental Table 1), raising concerns of placental insufficiency.

### Viral tropism and inflammatory response.

Because C57BL/6 mice are immune-complete and capable of producing interferon (IFN) and antibodies to clear the VSV-EBOV infection, the viral challenge in pregnant dams was expected to induce a limited and transient infection, followed by rapid systemic viral clearance, as previously observed in non-pregnant adult mice (14). Consistent with this expectation, no replicating virus was detected in the uterus, ovary, spleen, eyes, brain, kidney, liver, heart, and blood of the dams, nor in the placenta, amniotic fluid, and fetus, at 5 dpc (Figure 2, A and B), and viral RNA was only detectable in spleen (Supplemental Figure 1A). These observations suggested that the slight increase in resorbed pups and early postnatal deaths in dams inoculated on E7 was not associated with extended exposure to live virus. Next, we investigated whether the increase in pregnancy and postpartum losses could be attributed to a proinflammatory effect of the transient VSV-EBOV infection (19). To assess this, we measured the levels of proinflammatory and antiinflammatory cytokines in serum and placenta tissues from pregnant dams at 2 and 5 dpc. In placenta, there were no statistically significant differences in mRNA levels for any of the markers we tested at 2 dpc; however, by 5 dpc, the levels of TNF-α, IL-2, IL-6, IL-18, and IL-10 were significantly increased (Figure 2C and Supplemental Figure 1B). In sera, there was no significant difference in IFN-γ levels (Supplemental Figure 1C). These findings suggested that exposure to VSV-EBOV, despite its being rapidly cleared, modified the cytokine milieu within the placenta.

### Impact of VSV-EBOV on placental architecture.

The mouse placenta consists of the maternal decidua basalis (DB) and fetal embryo-derived compartments, including the junctional zone (JZ) and labyrinth zone (LB) (20). These structures are essential for fetal growth and survival by facilitating oxygen, nutrients, and waste exchange (21). Notably, the LB is composed of cells originating from both trophectoderm and mesoderm, which undergo branching morphogenesis to create a large surface area for maternal-fetal nutrient and gas exchange (22). Inflammation has been linked to placental dysfunction and poor maternal vascular adaptation (23); therefore, we next examined the structural integrity of trophoblast layers within the placental architecture of VSV-EBOV–vaccinated dams. At 5 dpc, placental weights in vaccinated dams were comparable to those in unvaccinated controls (Figure 3A). Histological analysis of placental tissues revealed no significant differences in the depth of DB, JZ, and LB between control and vaccinated groups, suggesting that VSV-EBOV vaccination was not associated with major placental architecture disruptions (Figure 3B). Next, we examined whether the viral challenge impacted glycogen trophoblast cells, which accumulate glycogen as a vital source of energy for placental and fetal development during early pregnancy and are present in the JZ of the mouse placenta until E12 (24). No significant differences were observed in the number of periodic acid–Schiff–positive (PAS^+^) glycogen cells in the JZ between vaccinated and control groups (Figure 3C), indicating that VSV-EBOV vaccination did not impact the glycogen storage in placenta. While these findings suggested that VSV-EBOV–induced placental inflammation did not affect decidualization, some statistically significant alterations were observed in the placenta from vaccinated dams. These included thickening of the spiral arterial (SpA) walls, characterized by narrow lumens and a cuffed appearance at 5 dpc (Figure 3D). In addition, vaccinated dams showed a significant reduction in mature *Dolichos biflorus* agglutinin–positive (DBA^+^) PAS^+^ vascular-associated uterine natural killer (va-uNK) cells in the DB, which are critical for guiding trophoblasts during SpA remodeling (Figure 3E). Notably, no significant differences were observed in the number of tissue-associated uNK cells in the DB between vaccinated and control groups (Figure 3E). Since inefficient SpA remodeling during pregnancy may be associated with delayed fetal growth, we next assessed fetal weight and developmental stages of the fetuses collected at 5 dpc (E12). As shown in Figure 3, F and G, there were no significant differences in fetal body weight or developmental maturity, as evaluated by Theiler stage (Figure 3H), between unexposed and VSV-EBOV–exposed groups at 5 dpc. Therefore, although there was a reduction of va-uNK cells with thickening of the arterial walls, placental weights and general architecture were conserved, and vaccination did not translate into evidence of delayed fetal growth.

### Impact of VSV-EBOV exposure on offspring development.

A critical feature of using an animal model to interrogate the impact of vaccination during gestation is that it allows assessment of whether there are long-term sequelae in the offspring of inoculated dams. To assess this, offspring born from dams challenged at E7 (*n* = 20), E10 (*n* = 24), or E15 (*n* = 13) were assessed for body weight gain and screened for signs of neurological and behavioral differences relative to unvaccinated mice (*n* = 25) at 6 months of age using the open field test (OFT), elevated plus maze (EPM), novel object recognition (NOR), and rotarod EPM tests. As shown in Figure 4A, the offspring from VSV-EBOV–exposed dams had a similar weight growth curve to those born from unvaccinated dams. However, 6-month-old female offspring born to dams vaccinated at E7, E10, and E15 displayed significantly higher body weight than offspring born to unvaccinated dams. In contrast, male offspring showed no changes in body weight (Figure 4B). No difference in distance, speed, freezing time, or travel to the center of the field was evident between groups in the OFT (Figure 4C). Similarly, there was no evidence of altered behavior in the EPM or the NOR test (Figure 4, D–F). However, the rotarod test, which measures motor function and coordination, showed a small but statistically significant reduction in latency time to fall among offspring from dams vaccinated with VSV-EBOV on E10 or E15 (Figure 4G). Since these differences were minor and not consistent for subgroups, our findings suggest that VSV-EBOV vaccination in C57BL/6 dams had no major long-term sequelae in the offspring, but additional studies may be warranted.

### Deficits in type I IFN signaling in pregnant dams led to increased viral load and higher rates of fetal resorption following VSV-EBOV exposure.

In previous studies, VSV-EBOV was observed to be rapidly cleared and did not cause symptoms or death in adult wild-type C54BL/6 mice (14). By contrast, VSV-EBOV was lethal in mice that were completely deficient in type I IFN signaling (14). To better understand the role of type I IFNs in the impact of VSV-EBOV vaccination in pregnant dams, we next used a complete or partial block of IFNAR with anti-IFNAR antibodies in mice receiving the vaccine. As shown in Supplemental Figure 2, mice that received a high dose of anti-IFNAR antibodies (1.8 mg/dam) died upon viral challenge. In contrast, administration of a sub-neutralizing dose of anti-IFNAR antibodies (0.6 mg/dam) 24 hours before challenge with 10^5^ TCID_50_ of VSV-EBOV on E7 enabled increased VSV-EBOV replication in C57BL/6 pregnant dams without increasing lethality (Supplemental Figure 2). To assess the role of type I IFNs, we next compared the outcomes of pregnant dams that were unvaccinated, vaccinated, or both vaccinated and treated with a low dose of anti-IFNAR 24 hours before vaccination (Figure 5A). No differences were observed in maternal body weight gain between the groups (Figure 5B); however, live viruses were detected in the uterus, ovary, and spleen as well as embryonic compartments including placenta, amniotic fluid, and remnants of reabsorbed fetuses in dams that were treated with anti-IFNAR before vaccination with VSV-EBOV (Figure 5C). Importantly, the susceptibility to infection in the anti-IFNAR antibody–treated vaccinated mice was dependent on EBOV-GP, since antibodies that block the glycoprotein protected the mice from VSV-EBOV replication (Supplemental Figure 3). Collectively, these findings suggested that type I IFN responses were critical to control the replication of VSV-EBOV following a subcutaneous challenge. The number of reabsorbed fetuses per pregnancy, as assessed by direct examination of the uteri at E12, increased in anti-IFNAR antibody–treated vaccinated dams compared with both unvaccinated and vaccinated dams (Figure 5D), which aligned with the presence of detectable virus in reabsorbed fetuses (Figure 5C). Interestingly, among the live fetuses derived from anti-IFNAR antibody–treated vaccinated mice, while the fetal body weight at E12 was not reduced (Figure 5E), which is consistent with the absence of viruses (Figure 5C), 3 of 16 fetuses showed higher Theiler stage (Figure 5F). This slight increase could correspond to the reduced number of fetuses per dam, which allows for increased nutrition and space.

The placental morphology in anti-IFNAR antibody–treated vaccinated dams was conserved (Supplemental Figure 4). Indeed, the placentas from anti-IFNAR antibody–treated vaccinated mice showed a similar number of glycogen cells in the JZ area, and normal SpA remodeling, in contrast to the abnormalities observed in VSV-EBOV–vaccinated dams (Figure 5, G and H). Both vaccinated dams and those treated with anti-IFNAR antibody showed significantly fewer mature va-uNK cells in the DB, but there were no significant changes in tissue-associated uNK cells in comparison with uninfected dams (Figure 5, I and J). Moreover, assessment of gene expression in placenta using a NanoString platform showed that the anti-IFNAR antibody–treated vaccinated mice had increased expression of interferon genes, but a reduction in immune cell markers (*Cd4*, *Cd8a*, *Itgax*, and *Pdcd1*) and costimulatory molecules (*Cd80*, *Cd69*, and *Fasl*) as well as relatively lower *Il6*, *Il18*, *Il17*, and *Lta* expression, compared with vaccinated dams (Supplemental Figure 5). These observations led us to hypothesize that while type I IFNs protected the dams from infection, they might contribute to the detrimental impact of VSV-EBOV vaccination. Previous studies suggested that IFN-β has a critical role in the maintenance of placenta integrity during infection (25, 26). To further explore whether VSV-EBOV–induced IFNs played a role in the mild increase in the number of resorbed fetuses and perinatal deaths, we administered VSV-EBOV to pregnant dams that selectively lacked IFN-β. As shown in Table 1, vaccination did not affect litter size, birth weight, or perinatal survival. Placentas from IFN-β–deficient vaccinated dams exhibited preserved structure, comparable glycogen cell numbers in the JZ, and normal SpA remodeling relative to unvaccinated controls (Figure 6, A–C). Notably, vaccination was associated with a significantly increased frequency of uNK cells in the DB and a significant reduction in va-uNK cells (Figure 6D). Despite these changes, the proinflammatory cytokine milieu was similar between vaccinated and unvaccinated IFN-β–deficient dams (Figure 6E). At E12, fetuses from vaccinated IFN-β–deficient dams showed a higher proportion of Theiler stage 20.5 compared with those from unvaccinated controls (Figure 6F). Together, these data indicate that type I IFN responses are critical to limit the replication of VSV-EBOV but may also contribute to the proinflammatory changes in placenta and have a detrimental impact on the offspring.

## Discussion

Clinical trials that involve replicating viruses usually exclude pregnant women, and this exclusion results in delays in acquiring information on how to best protect this important population in the event of an outbreak or travel to an endemic area (27, 28). Animal models that examine the potential impact of a vaccine on pregnant dams and their offspring can help assess the immediate and delayed risk of vaccinating pregnant and lactating women and aid in the decision of whether it is appropriate to include pregnant women in clinical trials. In this study, we showed that vaccinated pregnant mice developed antibody titers similar to those in non-pregnant females and cleared the virus by 5 dpc. While there was no evidence of live virus in viable fetuses and no apparent impact on litter size or birth weights, early vaccination (E7 peri-implantation) was associated with a small but significant increase in fetal resorption and early postnatal deaths. Examination of the placenta at 5 dpc showed a small but significant increase in proinflammatory cytokines in placenta, evidence of improper SpA remodeling, and reduced va-uNK cells in placenta. Notably, the absence of virus in viable fetuses was evident even in dams that had been treated with monoclonal antibodies that blocked type I IFNs despite the dams having higher viral loads. Moreover, the offspring of pregnant dams lacking IFN-β and vaccinated on E7 showed no evidence of increased resorptions or early postnatal deaths, suggesting that while type I IFNs are critical for controlling viral replication and maternal survival, they could contribute to the observed increase in detrimental proinflammatory placental responses.

Since 2014, VSV-ZEBOV vaccine has undergone extensive clinical studies to evaluate its safety and efficacy, including Partnership for Research on Ebola Vaccines in Liberia (PREVAIL; NCT02344407, ClinicalTrials.gov), Sierra Leone Trial to Introduce a Vaccine Against Ebola (STRIVE; NCT02378753), and a cluster-randomized ring vaccination trial in Guinea (Ebola Ça Suffit!; PACTR201503001057193). These clinical trials initially aimed to exclude pregnant and lactating women, and they were not included in trials until 2019. To date, published data regarding the vaccine’s outcomes in pregnant women are limited to a study that followed 84 women who were unintentionally vaccinated in early pregnancy or became pregnant after vaccination. This study found that 45% of women who were unintentionally immunized in early pregnancy experienced pregnancy loss, compared with 33% of unvaccinated women, and no congenital abnormalities were reported among the 44 live births (8). However, the sample size was insufficient to draw definitive conclusions on the impact of the vaccine (8). A new trial involving 840 women was initiated (28), but data from this larger cohort, which began vaccination in October 2019, are not yet available. An interesting observation from our study is that the adverse impact of VSV-EBOV vaccination was selectively observed at the peri-implantation period, when the trophoblast giant cells assume an invasive phenotype, thereby opening subluminal endometrial capillaries and establishing contact with maternal blood (29). This observation is consistent with previous studies in mice modeling the safety of another replicating virus vaccine for yellow fever, which also showed increased susceptibility at the time of implantation (30). Whether the increased risk is due to virus reaching the placenta or secondary to the proinflammatory response associated with the virus clearance is unknown. Future studies will be needed to determine whether vaccination later in gestation also induces systemic inflammatory cytokine production and whether the magnitude or kinetics of these responses differs by gestational stage. While the functional and structural organization of the maternal-fetal barrier in its early stages differs between humans and mice, our findings suggest the need to assess the clinical impact of vaccination in the peri-implantation period. Notably, implantation occurs at about days 8–9 in humans, so losses in humans are rarely detectable, which may hinder systematic assessment of impact on early pregnancy losses in women.

The placenta is a unique immunologic organ that is continuously remodeling and adapting to establish and maintain a maternal-fetal interface that protects the fetus against dangerous signals while inducing specific tolerance to paternal antigens. This balance relies on a local network of pro- and antiinflammatory cytokines. It is well established that when this barrier is breached, the lack of mature blood-brain barrier and immune system renders developing fetuses susceptible to viral meningoencephalitis, which can cause profound neurological damage (31). The increased susceptibility to viral infections early in life is also well documented in neonatal mice (32–34). Consistent with this, while adult mice control replication of VSV-EBOV, neonatal mice develop severe acute meningoencephalitis, retinitis, and death, which do not fully reproduce the human disease but can be used to test the efficacy of therapeutics in vivo (14–16). Interestingly, no live virus was evident among the live offspring of pregnant mice that were challenged with VSV-EBOV, regardless of challenge time or whether the tissues were recovered from the fetus in utero or from the newborns after births. Indeed, there was no evidence of live virus in the offspring, even in dams with reduced type I IFN–mediated protection, which resulted in extensive viral penetration in other tissues as evidenced by high virus titers in placenta and amniotic fluid. Furthermore, the effects appear stochastic, as most fetuses within each litter progressed to full term without adverse outcomes. Notably, at 11 dpc (E18), infectious virus was no longer detectable in the uterus, ovary, spleen, or amniotic fluid, and similarly, no virus was recovered from P0 live pups born to anti-IFNAR–treated vaccinated dams (Supplemental Figure 6). In contrast, remnants of resorbed fetuses showed high viral titers. The presence of virus in the fetus remnants but not in live fetuses suggests that the intact placenta can insulate the fetus from hematogenous infection. Alternatively, fetally derived type I IFNs, which would not be blocked by the anti-IFNAR antibodies since murine IgGs do not cross the mouse placenta (35), may be sufficient to confer protection. Placentally derived type I IFNs may also contribute to preventing fetal infection. Notably, there was one exception (Supplemental Figure 6), where a fetus recovered apparently intact at 11 dpc (E18) exhibited high viral titers; it is unclear whether this fetus was viable and its placental barrier intact at the time. Additional studies will be needed to determine whether a failure in the placental barrier permits direct viral transmission to the fetus, leading to fetal death via cytopathic effects, or whether fetal demise initiates retrograde placental degradation and infection of fetal tissue remnants. Regardless of the underlying mechanism, our data indicate that even if VSV-EBOV were to cause transient maternal viremia or reach the placenta, the risk of vertical transmission of this attenuated virus is low, particularly in late gestation.

In our study, we examined the impact of the transient infection with VSV-EBOV on the integrity of the placenta. It is known that altered LB to JZ ratio can limit the supply of nutrients and oxygen to the fetus, thereby restricting fetal growth (36). Previous studies show that Zika virus (ZIKV) activates type I IFN signaling in the placenta, which delays labyrinth development and disrupts the structure of the maternal-fetal barrier, leading to abnormal placental architecture with adverse consequences for the fetus (37). In this study, VSV-EBOV vaccination did not decrease labyrinth/junctional zone ratio in wild-type dams or anti-IFNAR antibody–treated vaccinated dams, in which placentas harbored high titers of VSV-EBOV. Interestingly, we found that blocking IFNAR ameliorated VSV-EBOV–induced inefficient SpA remodeling despite the presence of viral infection in the placenta. These findings are in agreement with those of Yockey et al. in a ZIKV pregnancy model (37), in which *Ifnar^–/–^* mouse fetuses progressed to term whereas the *Ifnar^+/–^* littermates did not, identifying type I IFNs as critical mediators of placental disruptions in prenatal viral infections. Together these findings suggest that antiviral responses result in increased type I IFN and proinflammatory cytokine levels and drive placental changes, highlighting the complex interplay between maternal immune modulation and placental function. Further studies with cell type–specific resolution will be needed to clarify how type I IFN influences placental vascular remodeling and fetal outcomes. We also investigated the impact of VSV-EBOV vaccination on NK cell populations at the feto-maternal interface, as these cells play a crucial role in SpA remodeling, fetal development, and maternal immune adaptation (38, 39). Previous studies have shown that decidual NK cells can become activated to defend against pathogen infections including ZIKV, human immunodeficiency virus, and human cytomegalovirus (39). Interestingly, we observed a decrease in mature vascular-associated uNK cells in both wild-type and IFNAR-suppressed pregnant dams following vaccination, while tissue-associated NK cells remained unchanged. The reduction in vascular-associated uNK cells likely contributes to impaired vascular remodeling and placental malperfusion (21, 40). It is important to note that one limitation of this study is the use of syngeneic C57BL/6 matings, whereas human pregnancy occurs in an allogeneic setting in which uNK cells and regulatory T cells contribute to fetal tolerance and placental development (41). Because allogeneic mating can shape immune composition and vascular remodeling at the maternal-fetal interface, our animal model may not fully capture these alloimmune interactions. Future studies using allogeneic matings may help determine whether vaccine-associated placental inflammation can also occur in this environment.

Importantly, infection and demise of the offspring is not the only consideration. Fetal inflammatory response syndrome is a condition in which, despite an absence of cultivable microorganisms, neonates with placental infections are exposed to increased levels of proinflammatory cytokines (42). This may affect the development of the CNS and imprint the fetal immune system. Indeed, a substantial body of literature suggests that even in the absence of infection, early inflammation insults that elicit a strong Toll-like receptor–mediated (TLR-mediated) activation, such as administration of TLR3 and/or TLR4 agonists that increase type I IFN levels, can result in abnormal behavior in the progeny resembling autism and schizophrenia (43). It is notable that the increase in prenatal and early postnatal losses was not evident in mice lacking IFN-β, which is a key regulator in placental immune system (44, 45). Previous studies in which pregnant mice received poly I:C have suggested that prenatal exposure to IFNs could be associated with neurological or psychiatric sequelae (46–48). Our study assessing the growth curve of these animals did not show a change in body weight gain over time or major overall differences in motor activity or behavior, but a reduced latency to fall in the rotarod test was evident among the offspring of vaccinated dams (49). Notably, the observed differences are subtle and not consistent between subgroups, suggesting that they could be stochastic. Thus, additional studies using a much larger number of animals and a more complete set of behavioral studies will be needed to understand whether there is long-term impact on behavior or cognitive ability. Although translating from behavioral studies in mice to humans is extremely challenging owing to significant biological and cognitive differences, it may be valuable to conduct these, as Ebola outbreaks and immunization campaigns often occur in areas where conflict hinders long-term follow-up of vaccinees (50).

Concerns about maternal and fetal safety often exclude pregnant women from clinical trials of live, replicating vaccines, leaving critical gaps in our understanding of their efficacy and risk during pregnancy (28, 51). This knowledge gap complicates risk-benefit assessments during outbreak responses. Preclinical models that evaluate vaccine safety in pregnancy can help address these uncertainties, although the interpretation of adverse outcomes requires careful contextualization given the critical differences in vaccine dose, route of administration, and viral replication kinetics, as well as placental architecture. Importantly, such models can be modulated to mimic worst-case scenarios, by enhancement of viral exposure, or protective conditions, such as by blocking of type I IFN signaling or administration of anti-EBOV antibodies, providing a versatile platform to study pathophysiology and potential interventions. Our findings suggest that peri-implantation VSV-EBOV vaccination could increase a risk of pregnancy loss; however, it remains unclear whether these effects represent an inherent consequence of vaccine-induced antiviral immunity or are potentiated by augmented type I IFN signaling under the experimental conditions employed. Despite these limitations on preclinical studies, the model described here offers a framework to improve our understanding of VSV-EBOV vaccines, to assess maternal-fetal risks, and to screen therapeutics that could mitigate adverse outcomes in this vulnerable population.

## Methods

Further information can be found in Supplemental Methods.

### Sex as a biological variable.

Both male and female offspring were included in this study. Sex was considered as a biological variable in the analyses, as vaccination during pregnancy had a significant effect on body weight specifically in female offspring, whereas no significant changes were observed in male offspring.

### Mice and tissue collection.

C57BL/6J mice from The Jackson Laboratory were bred and housed in sterile microisolator cages under a 12-hour day/12-hour night cycle in the specific pathogen–free, Association for Assessment and Accreditation of Laboratory Animal Care International–accredited animal facility of the US Food and Drug Administration’s (FDA’s) Division of Veterinary Medicine. C57BL/6 mice deficient in *Ifnb1* (*Ifnb1^–/–^*) were provided by Stefanie Vogel (Department of Microbiology and Immunology, University of Maryland School of Medicine, Baltimore, Maryland, USA) (52). All females were between 3 and 6 months of age at the time of pregnancy, and any offspring were weaned 21 days after birth and housed with same-sex littermates (2–5 mice per cage). Mice were given water and commercial 5P76 Prolab Isopro RMH 3000 feed (Lab Diet, catalog 0006972) ad libitum.

### Timed pregnancies.

Timed pregnancies were initiated by pairing of a single female with an age-matched male overnight. The pair was separated at noon the next day and the time denoted as embryonic day 0 (E0). On E6, mice were anesthetized with isoflurane (2% induction, 1.5% maintenance dose) in oxygen (1.5 L/min). Fur was removed from the abdomen with a depilatory cream, and then the mouse was transferred to a heated platform where pre-warmed ultrasound transmission gel was applied. The presence of embryos was confirmed using the FUJIFILM VisualSonics VEVO 2100 imaging system (FUJIFILM VisualSonics). Maternal respiratory rate was monitored for depth and regularity.

### Pseudotyped viruses.

The VSV-G–deficient vesicular stomatitis virus expressing the Zaire (Mayinga) Ebola virus glycoprotein (VSV-EBOV) was obtained from BEI Resources, supported by the National Institute of Allergy and Infectious Diseases and managed by ATCC (catalog NR-29021). Viral propagation was carried out in VERO E6 cells to generate a master stock, which was subsequently preserved at –80°C. The viral titer of the produced stocks was determined using the TCID_50_ assay following the protocol described by McWilliams et al. (14).

### Vaccination and serum collection.

Viral stocks were thawed before each vaccination and diluted to working concentration to administer a single dose of 1 × 10^5^ TCID_50_ of virus in 50 μL sterile phosphate-buffered saline (PBS; Gibco, catalog 10010049) subcutaneously in the lower right abdominal quadrant of pregnant mice. The challenge was administered as a single dose on either E7, representing the early-gestation group; E10 for the mid-gestation group; or E15 for the late gestation group. Control mice received a bolus of 50 μL of sterile PBS on the corresponding gestational day for each group. Body weights of mice were collected at 3 and 5 days after challenge or PBS treatment for the E7 and E10 study groups, and on day 3 for the E15 group. Body weight change was calculated as the change in mouse weight over time for each experiment [(mouse weight at day X post-vaccination – weight on day of vaccination) / (weight on day of vaccination)] × 100. Blood samples were collected by tail-vein bleed before pregnancy and at 21 and 42 days post-challenge (dpc) for sera and stored at –20°C for antibody measurements.

### Antibody titer ELISA.

Immulon 2 HB flat-bottom plates (96-well; Thermo Fisher Scientific, catalog 3455) were coated with 200 μL of 1 × 10^6^ TCID_50_/mL of VSV-EBOV in Opti-MEM I medium (Gibco, catalog 31985070). Virus-coated plates were incubated with mouse serum samples overnight at 4°C, followed by secondary antibodies against mouse IgG (SeraCare, catalog 074-18-061). The signals were read with a VICTOR X Light Luminescence Plate Reader (PerkinElmer) at 450 nm.

### Offspring survival.

Cage-side observations were performed daily beginning on E16 to collect offspring body weight within 3 hours of parturition. Offspring weight was calculated by averaging of the individual weights within each litter, followed by calculation of the group average. Offspring survival was monitored until P14, after which survivors were euthanized by CO_2_ asphyxiation. A subset of offspring was maintained and monitored for behavioral studies beginning on P60.

### Tissue collection.

A subset of E7-vaccinated or PBS-treated dams were euthanized at 5 dpc (on E12) by CO_2_ asphyxiation and exsanguinated by transcardiac perfusion with ice-cold PBS before collection of tissue for RNA extraction, viral quantification by TCID_50_, or histology. E12 was selected to capture early fetal and placental changes following maternal vaccination, before the optimal time for visualizing abortion rates in mice. The uterine horn was exposed and examined for abnormal implantation sites, followed by removal and immersion in ice-cold PBS. The percentage of reabsorbed fetuses was calculated (non-viable implantations / total implantation × 100), and individual concepti (embryo, placenta, and surrounding uterus) were separated and alternatingly allocated whole to histology or for processing as isolated specimens for uterine tissue, placenta, amniotic fluid, or fetus. Isolated placentas were further separated into 2 sections to perform RNA extraction and TCID_50_ viral quantification in parallel. Only viable concepti were allocated for specimen analysis, and tissue was flash-frozen and stored at –80°C.

### Fetal body weight and Theiler stage analysis.

Fetuses isolated at E12 were fixed in Bouin’s fixative (LabChem, catalog 0178-LC117901) and subsequently cleared in 75% vol/vol ethanol for body weight measurement and Theiler stage analysis. Weights were obtained by air-drying of the fetuses (every 30 seconds for 8 minutes due to alcohol volatilization), and fetal developmental stages were determined according to Theiler stage criteria (http://www.emouseatlas.org/emap/home.html). Theiler stages were evaluated under a Leica S6E microscope (Leica), and fetal images were obtained using iPhone 11 Pro camera (Apple).

### Placenta morphology characterization.

Formalin-fixed and paraffin-embedded placentas were serially sectioned and subjected to hematoxylin and eosin (H&E) staining. Stained slides were scanned by Olympus system and OlyVIA software. Morphometric analysis was conducted to assess the composition of placenta layers (labyrinth zone [LB], junctional zone [JZ], and decidua basalis [DB]). The measurements were performed 3 times in each section, and the average of the results was calculated. A vertical line was drawn from the maternal gland to the chorionic plate at the placenta’s central region, with subsequent measurement of the length of each layer (in micrometers). In addition, the H&E staining result was also used to investigate the hemorrhagic region in DB. The measurement of each hemorrhagic site within the DB layer, as well as the DB area, was calculated as ratio between the total hemorrhagic area and DB area.

### Maternal spiral artery remodeling.

Masson-Goldner staining was conducted on the paraffin slides, as described previously (21, 53), to evaluate the thickness of the maternal spiral artery (SpA) walls. Briefly, placenta samples were Masson-Goldner stained, scanned, and analyzed using the Olympus system. For each analyzed placenta, we measured the 5 largest and roundest SpAs, including the total vessel diameter (μm) and lumen diameter (μm) separately. To assess the wall thickness, the ratio of vessel diameter to lumen diameter was calculated.

### Spatial NK cell analysis.

*Dolichos biflorus* agglutinin (DBA)/periodic acid–Schiff (PAS) staining was performed and analyzed on E12 placenta as previously described (54). Briefly, the sections were then scanned and analyzed with the Olympus system followed by ImageJ (v1.53a, NIH) software. The quantification of tissue- and vascular-associated uNK cells was conducted separately in the DB. In each implantation, 5 sections were scored for number of uNK cells and normalized by 1 square millimeter.

### Glycogen cells in the outer zone of the placenta.

We investigated the placental morphology of the different experimental groups at E12 using PAS. The area occupied by glycogen cells in the JZ and the ratio of glycogen cells in the outer zone were calculated by ImageJ (v1.53a) software as previously described (55). The PAS^+^ glycogen trophoblast cells were identified using manual thresholding. The minimum threshold was fixed at 10, while the maximum threshold ranged from 80 to 105 depending on staining intensity. Quantification was performed in a blinded manner, and the same maximum threshold was applied with comparable staining intensity.

### Cytokine profiling.

Placental tissues were homogenized in PBS (Gibco, Thermo Fisher Scientific, catalog 14190144) and centrifuged at 6,000*g* for 10 minutes at 4°C. Supernatants were transferred to clean microcentrifuge tubes and stored at –80°C until further analysis. Cytokines in placenta homogenates were measured using the Mouse Th1/Th2/Th9/Th17/Th22/Treg Cytokine 17-Plex ProcartaPlex Panel (Invitrogen, catalog EPX170-26087-901) and the Luminex xMAP (multianalyte profiling) technology (Luminex Corporation, a DiaSorin company) as previously described (56). Results were normalized by the protein concentration tested by Bradford assay.

### Cell lines.

The VERO E6 kidney epithelial cell line (ATCC, catalog CRL-1586) was cultured in Minimum Essential Medium (MEM; Gibco, catalog 11095080) supplemented with 10% fetal bovine serum (R&D Systems, catalog S11550), 1% penicillin-streptomycin (Thermo Fisher Scientific, catalog 15140163), and 1% l-glutamine (Gibco, catalog 25030081). Cells were maintained at 37°C in a humidified incubator with 5% CO_2_. According to ATCC, the sex of this cell line has not been specified.

### TCID_50_ viral quantification.

VERO E6 cells were seeded into 96-well plates at 100 μL per well using complete MEM, aiming for 70%–90% confluence after 24 hours of incubation. Tissue samples were homogenized, and the homogenates were clarified by centrifugation to remove debris. The resulting supernatant was serially diluted in MEM without supplements, and 100 μL of each dilution was transferred onto the VERO E6 monolayers. Cytopathic effects were evaluated 4–5 days after inoculation, and the TCID_50_ values were determined following established procedures (34).

### Behavioral tests.

Behavioral testing was performed following previously established procedures (15). In brief, the open field test (OFT) was used to evaluate spontaneous locomotion and anxiety-related behavior. Each mouse was placed in one corner of a square arena (40 × 40 cm) and allowed to move freely for 30 minutes. Activity was recorded with an overhead camera, and parameters such as total distance traveled and time spent in the central zone (inner 50% of the arena) were quantified using ANY-maze software (Stoelting Co.).

Anxiety-like behavior was further examined using the elevated plus maze (EPM). The apparatus consisted of 2 open arms (39.5 × 5 cm) and 2 perpendicular enclosed arms (35 × 5 × 15 cm) extending from a central platform (5 × 5 cm), elevated 50 cm above the floor. Mice were placed in the central area facing an open arm and allowed to explore for 10 minutes while their movements were recorded by an overhead camera. Time spent in open and closed arms and total distance traveled were automatically analyzed using ANY-maze.

For the novel object recognition (NOR) test, mice were preconditioned 1 day earlier by being placed in the open-field arena with 2 identical objects (either black spheres or white cubes). Mice were allowed to freely explore the objects for 10 minutes before being returned to their home cages. On the following day, one of the familiar objects was replaced with a novel object. Mice were then reintroduced to the arena for a 5-minute test session. Behavior was recorded using an overhead camera, and time spent interacting with familiar versus novel objects was automatically analyzed using TopScan software (CleverSys) or ANY-maze software.

Motor coordination and balance were assessed using the rotarod test. Mice were placed on a rotating drum (3.2 cm diameter) that gradually accelerated from 5 to 20 rpm over a 3-minute period. The latency to fall from the rod was recorded across 3 trials, and the mean value was used for subsequent analysis. A cutoff time of 180 seconds was applied if the mouse remained on the rod for the full duration.

### Statistics.

Comparisons between groups used the 2-tailed unpaired Student’s t test, Mann-Whitney U test, ANOVA and Kruskal-Wallis test as appropriate (indicated in each figure legend); *P* values were adjusted for multiple comparisons as appropriate. Statistical analyses were conducted with GraphPad Software (version 10.0.0). Adjusted *P* < 0.05 was considered statistically significant.

### Study approval.

The experimental protocol 2016-13 was reviewed and approved by the FDA Animal Care and Use Committee (FDA-ACUC), and animal practices used in these studies conformed to relevant regulatory standards. All procedures were performed in accordance with the FDA-ACUC guidelines.

### Data availability.

Values for all data points are included in the Supporting Data Values file.

## Author contributions

APL, HNL, SMB, and DV designed the study. APL and YW mainly performed the experiments. APL, HNL, and DV analyzed the data. YW and SMB performed immunohistochemistry and analyzed the images. MIM, KE, IR, ILM, DDCI, and MM helped with the experiments. AL helped with ultrasound. LKB helped with statistical analysis. APL, YW, HNL, DV, and SMB discussed the data. APL and HNL wrote the manuscript. DV and SMB revised the manuscript. All authors read and approved the final version of the manuscript.

## Conflict of interest

The authors have declared that no conflict of interest exists.

## Funding support

- US FDA Perinatal Health Center of Excellence grant.
- Postgraduate Research Fellowship Awards from the Oak Ridge Institute for Science and Education through an interagency agreement between the US Department of Energy and the US FDA to DV.
- Deutsche Forschungsgemeinschaft grants through the Heisenberg Program (BL1115/11-1, SFB1713 project 534829736) to SMB.
- China Scholarship Council Program to YW.

## Supplementary Material

Supplemental data

Supporting data values

## Figures and Tables

**Figure 1:**
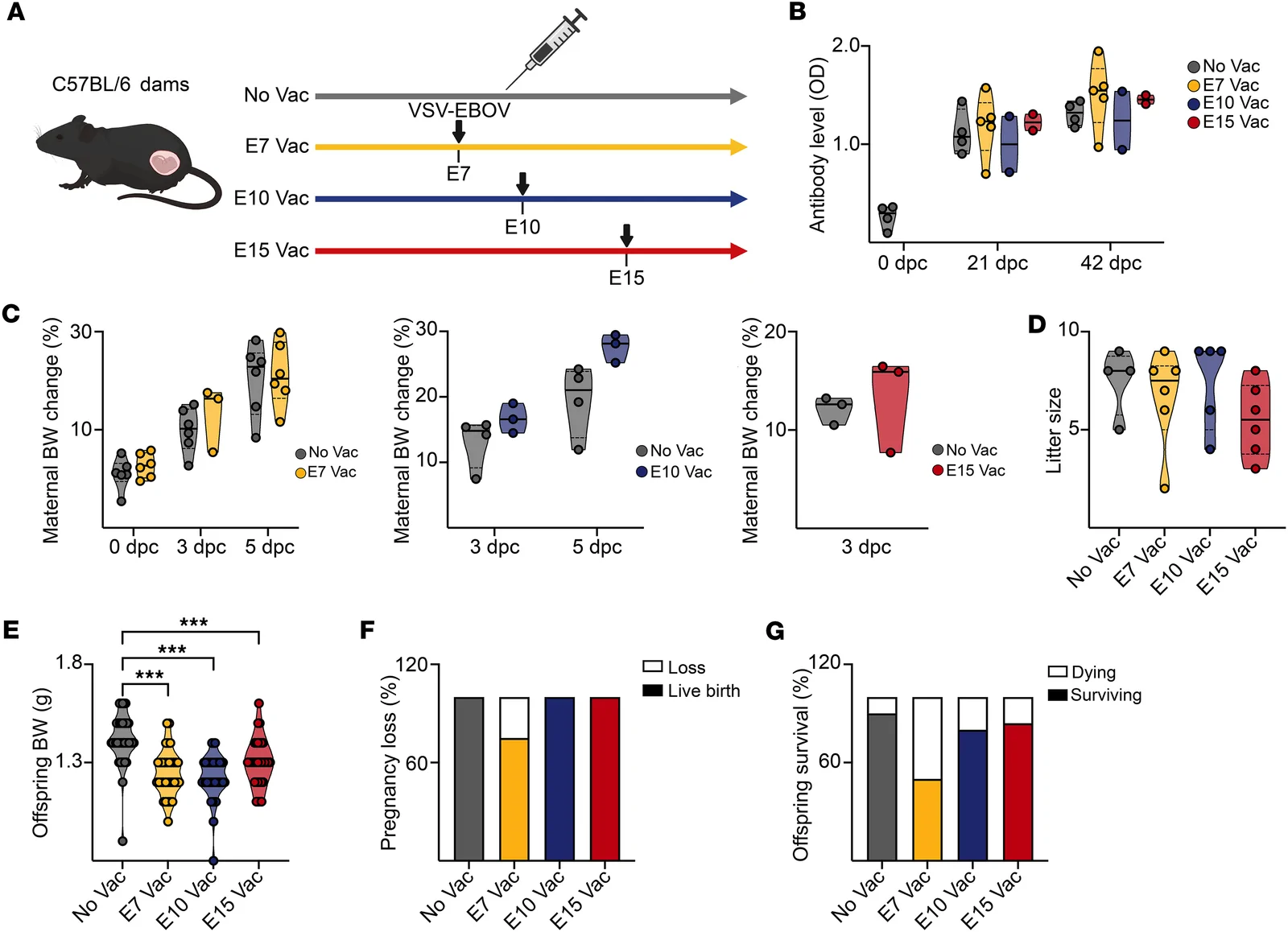
Vaccination of pregnant C57BL/6 mice with VSV-EBOV at distinct gestational stages. (**A**) Schematic of the experimental design. Pregnant C57BL/6 mice were vaccinated (Vac) subcutaneously with 10^5^ TCID_50_ VSV-EBOV at embryonic day7 (E7; early gestation), E10 (mid-gestation), or E15 (late gestation). (**B**) Anti–VSV-EBOV antibody titers measured by ELISA at 0, 21, and 42 days post-challenge (dpc). Data are presented as mean ± SD (*n* = 2–5 mice per group). (**C**) Maternal body weight (BW) changes in pregnant mice with or without vaccination at E7, E10, or E15. Body weight change was calculated as [(weight at day X post-vaccination – weight at day 0) / weight at day 0] × 100. Data are presented as mean ± SD (*n* = 3–6 mice per group). (**D** and **E**) Litter size (**D**) and offspring body weight at birth (**E**) from unvaccinated dams or dams vaccinated at E7, E10, or E15. (**F** and **G**) Percentage of fetal loss during pregnancy (**F**) or offspring survival (**G**). Statistical significance was determined by 2-way ANOVA for **B** and **C** (left and middle), Mann-Whitney *U* test for **C** (right), and Kruskal-Wallis test followed by Dunn’s multiple-comparison test for **D** and **E**. ***P < 0.001.

**Figure 2:**
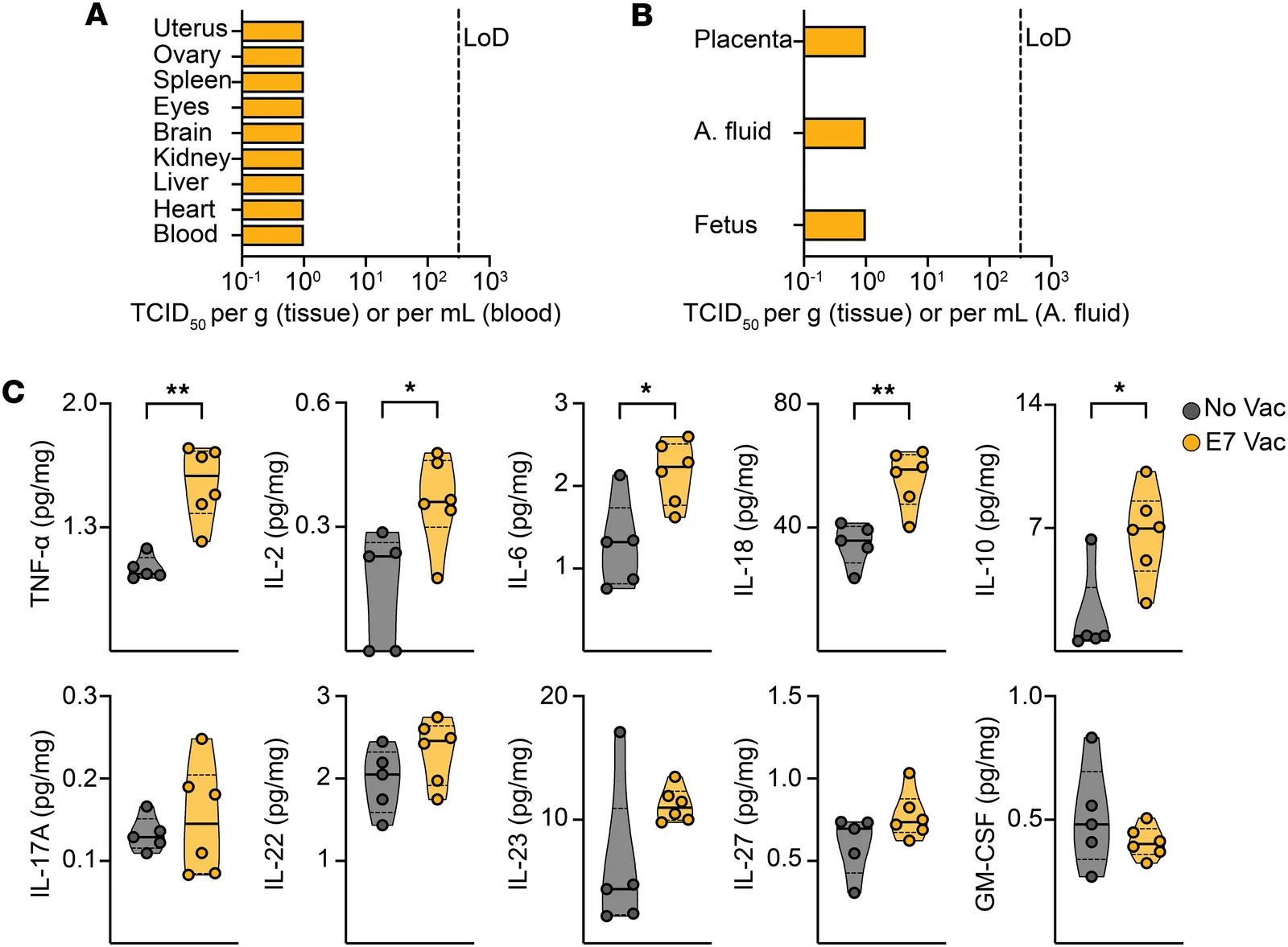
Early-gestational vaccination with VSV-EBOV induces proinflammatory cytokine responses in the placenta. Pregnant C57BL/6 mice were vaccinated with 10^5^ TCID_50_ VSV-EBOV at E7 and sacrificed at 5 dpc (E12). Unvaccinated control (No Vac) dams were collected at E12. (**A** and **B**) Viral titers in maternal tissues (**A**) and embryos (**B**) were quantified by TCID_50_ assay. The dashed lines indicate the limit of detection (LoD = 316 TCID_50_). (**C**) Placental protein levels of TNF-α, IL-2, IL-6, IL-18, IL-10, IL-17A, IL-22, IL-23, IL-27, and GM-CSF were measured in lysates from control and VSV-EBOV–vaccinated dams at 5 dpc using a Luminex assay. Data are presented as mean ± SD (*n* = 5–6 per group). *P < 0.05, **P < 0.01 vs. control placentas, as determined by 2-tailed Mann-Whitney *U* test. A. fluid, amniotic fluid; TCID_50_, 50% tissue culture infectious dose.

**Figure 3:**
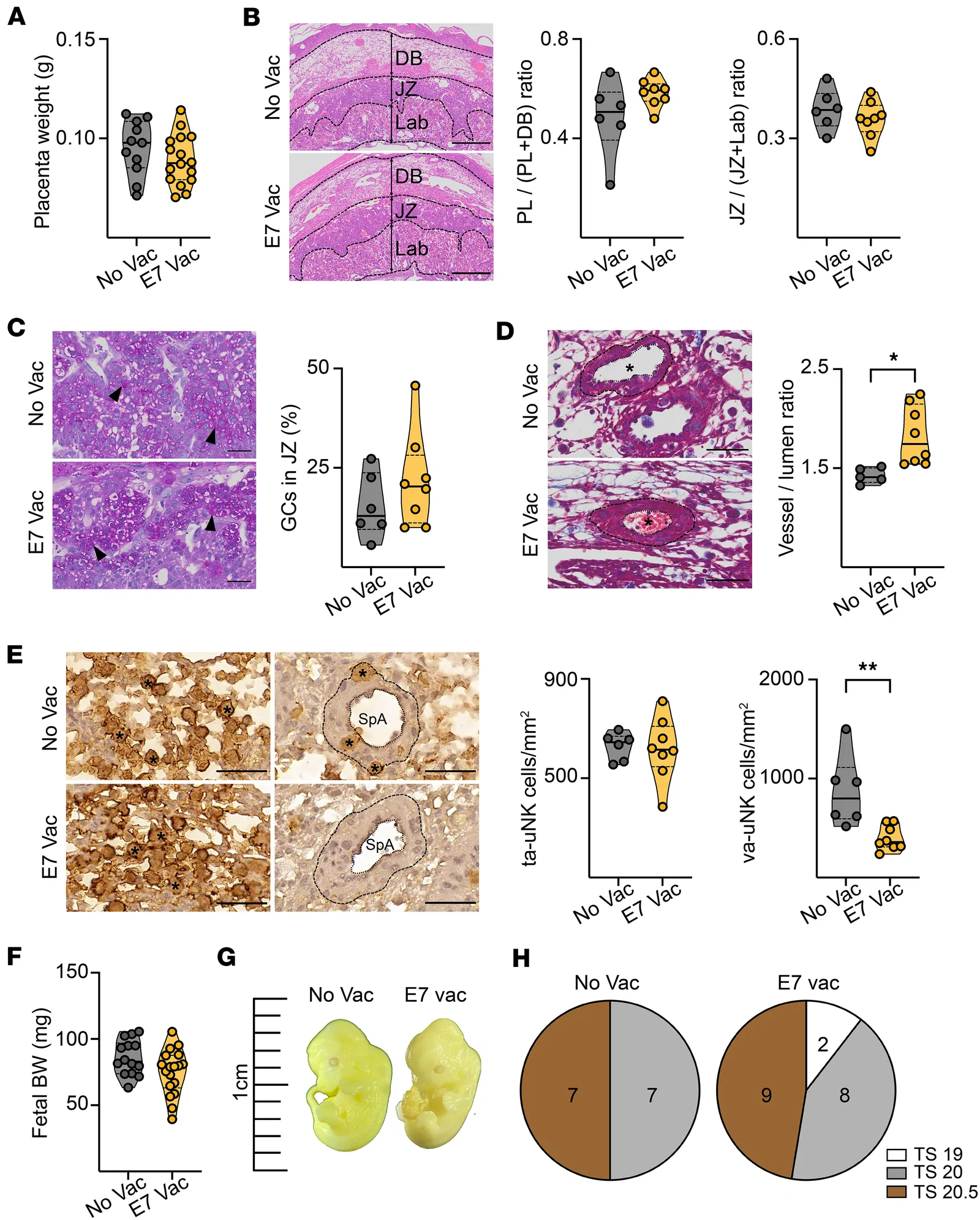
VSV-EBOV vaccination in pregnant dams affects placental morphology but does not impair fetal development. Pregnant C57BL/6 mice were challenged with 10^5^ TCID_50_ VSV-EBOV at E7 and sacrificed at 5 dpc. (**A**) Placental weight was measured at E12. Data are shown as mean ± SD (*n* = 11–16 per group). (**B**) H&E staining of E12 placental sections from unvaccinated (No Vac) and vaccinated (E7 Vac) dams. Quantification shows the relative proportion of placental labyrinth (PL) depth to combined PL and decidua basalis (DB) depth (left), and junctional zone (JZ) to combined JZ and labyrinth (Lab) depth (right). Scale bars: 500 μm. (**C**) PAS staining of E12 placental sections to assess glycogen-rich cells. Arrowheads indicate glycogen cells (GCs). Scale bars: 50 μm. The graph shows the proportion of PAS^+^ cells within the JZ. (**D**) Masson-Goldner trichrome staining of E12 placental sections. Black asterisks indicate spiral arteries (SpAs). Scale bars: 50 μm. The graph shows SpA outer-to-luminal diameter ratio. Measurements in **B**–**D** were performed in triplicate. (**E**) *Dolichos biflorus* agglutinin (DBA)/PAS staining of E12 placental sections showing accumulation of mature uNK cells. Double-positive (DBA^+^PAS^+^) tissue-associated uNK (ta-uNK; left) and vascular-associated uNK (va-uNK; right) cells are denoted by black asterisks. Scale bars: 50 μm. Five fields per sample were analyzed; counts were normalized to 1 mm^2^. (**F**–**H**) Fetal parameters at E12: weight (**F**) and Theiler stage (TS) (**G** and **H**), independently assessed by 2 investigators on 2 occasions. Data are shown as mean ± SD (n = 14–19 per group) and up to 8 per group unless otherwise indicated *P < 0.05, **P < 0.01 vs. control dams, as determined by unpaired 2 tailed Student’s *t* test.

**Figure 4:**
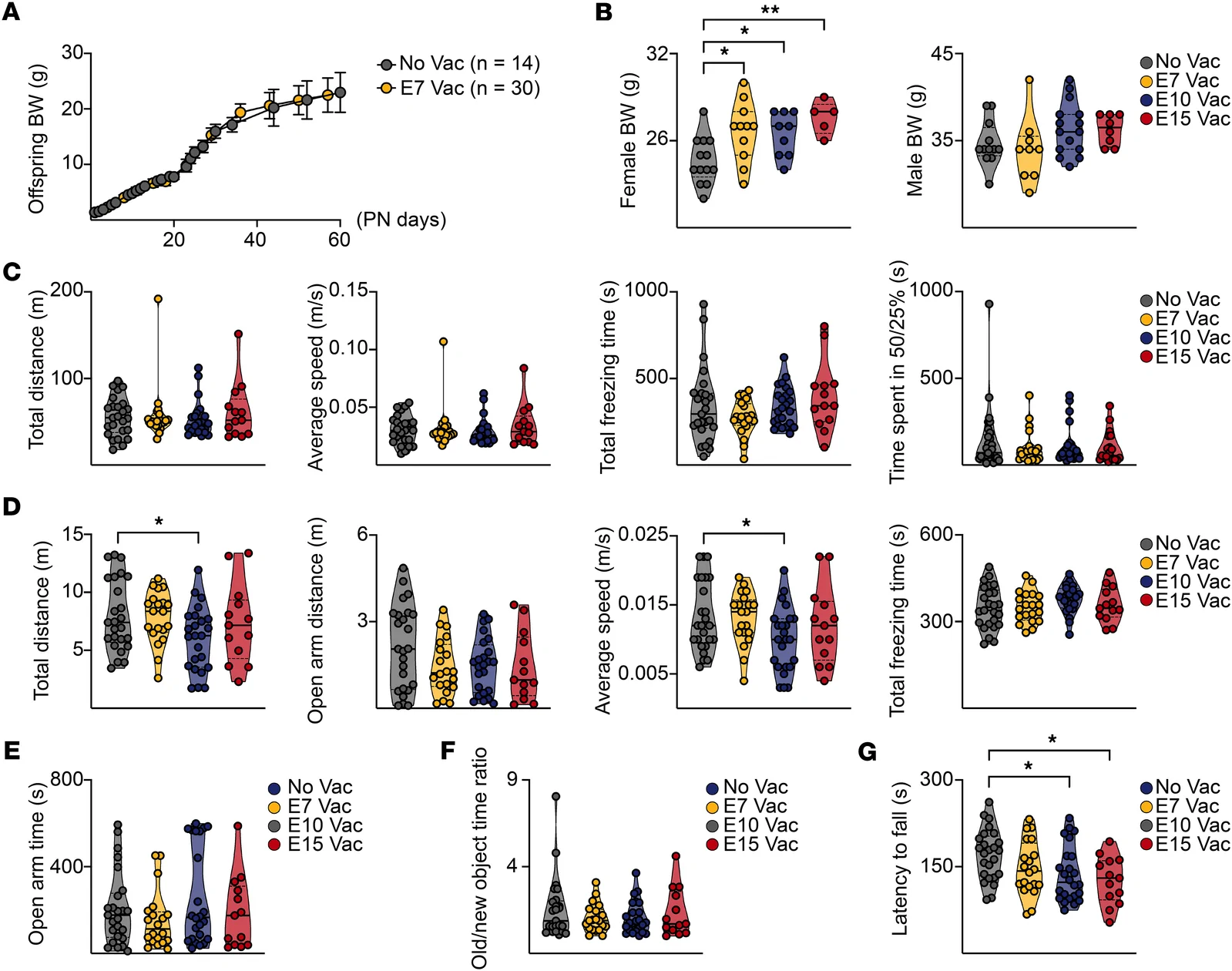
Offspring born to VSV-EBOV–vaccinated dams display comparable growth and behavioral outcomes. (**A**) Offspring born to unvaccinated (No Vac) dams (*n* = 14) or dams vaccinated at E7 (E7 Vac) (*n* = 30) were monitored for body weight changes over 60 days. PN, postnatal. (**B**–**G**) Female and male 6-month-old offspring born to unvaccinated (No Vac) dams or dams vaccinated at E7 (E7 Vac), E10 (E10 Vac), or E15 (E15 Vac) were assessed for body weight (**B**) and behavioral performance (**C**–**G**). (**C**) Open field test: total distance traveled, average speed, total freezing time, and time spent in the center 50% and 25% of the arena. (**D** and **E**) Elevated plus maze: total distance traveled, distance traveled in open arms, average speed, total freezing time, and time spent in open arms. (**F**) Novel object recognition test: interaction ratio (novel vs. familiar object), calculated as time spent with novel relative to familiar objects. (**G**) Rotarod test: average latency to fall (mean of 3 trials per mouse) on an accelerating rotating rod over 180 seconds. Data are shown as mean ± SD (*n* = 5–25 per group). *P < 0.05, **P < 0.01 vs. offspring from unvaccinated dams, as determined by 1-way ANOVA followed by Dunnett’s multiple-comparison test.

**Figure 5:**
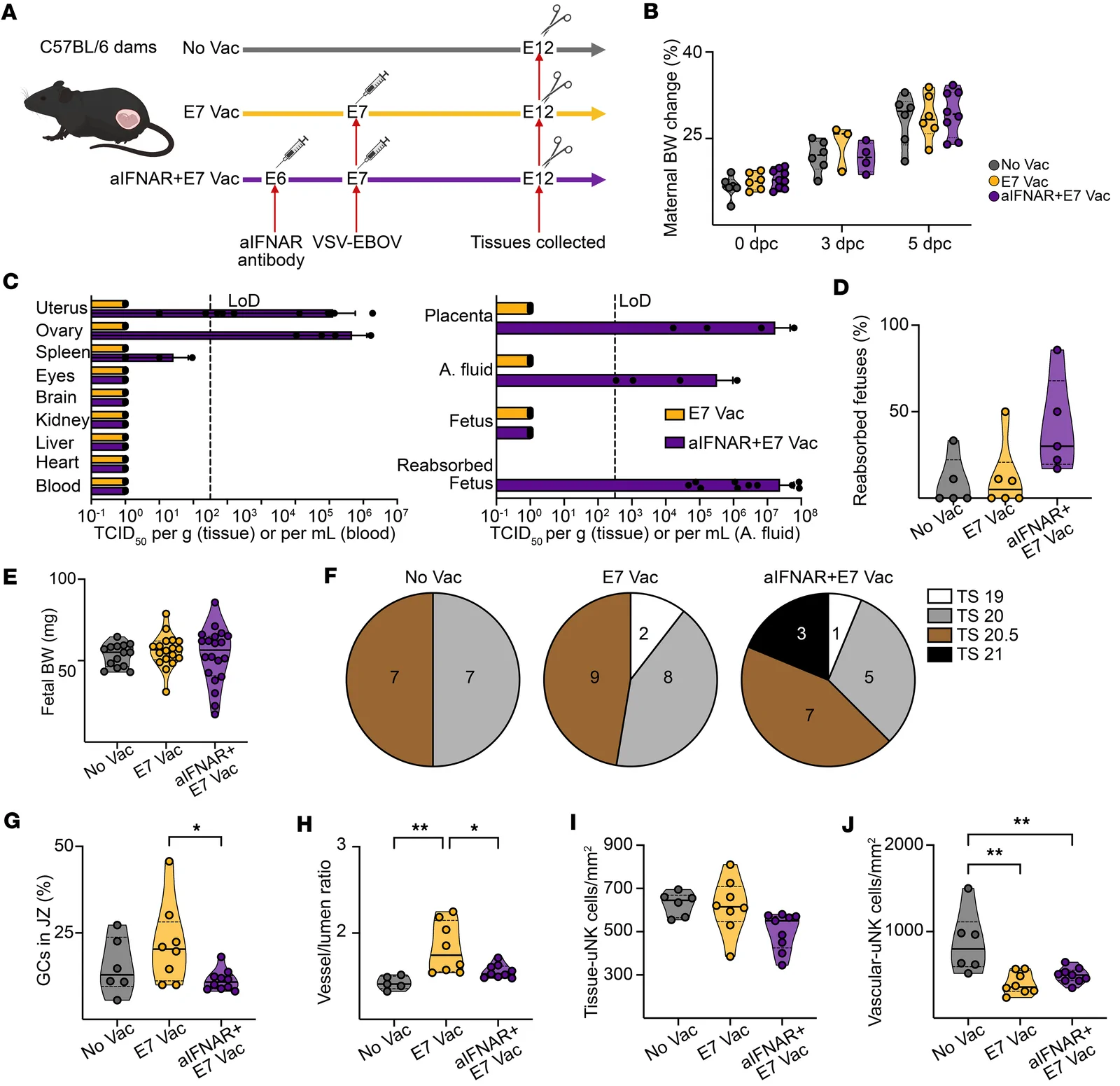
Blockade of type I IFN signaling in vaccinated pregnant dams increases viral load and fetal resorption while attenuating vaccine-induced placental alterations. (**A**) Schematic of the experimental design. Pregnant C57BL/6 mice received anti-IFNAR antibodies (aIFNAR; 0.6 mg, i.p.) at E6 and were challenged with 10^5^ TCID_50_ VSV-EBOV at E7, followed by sacrifice at E12. Uninfected control pregnant C57BL/6 mice (No Vac) were sacrificed at E12. (**B**) Maternal body weight (BW) was monitored at E6 (baseline), 3 dpc, and 5 dpc. Weight changes are shown relative to E6. Data are presented as mean ± SD (*n* = 3–9 mice per group). (**C**) Viral loads in dams and embryos were quantified by TCID_50_ assay. Dashed lines indicate the limit of detection (LoD = 316 TCID_50_). (**D**–**F**) Fetal outcomes at E12, including the rate of resorbed fetuses (**D**), fetal body weight (**E**), and developmental stage assessed by Theiler staging (TS) (**F**) in the indicated groups. Data are shown as mean ± SD. Number of fetuses per group is indicated within the pie charts. (**G**) Glycogen-rich cells were assessed by PAS staining. The graph shows the proportion of PAS^+^ cells within the junctional zone (JZ). GCs, glycogen cells. (**H**) SpA remodeling was evaluated by Masson-Goldner trichrome staining. (**I** and **J**) DBA/PAS staining of E12 placental sections showing accumulation of mature uNK cells. Double-positive (DBA^+^PAS^+^) tissue-associated uNK (**I**) and vascular-associated uNK (**J**) cells. In **G**–**J**, data from control (No Vac) and vaccinated (E7 Vac) groups are shown again for reference alongside the anti-IFNAR–treated vaccinated (aIFNAR + E7 Vac) group. Data are presented as mean ± SD (*n* = 6–9 per group). *P < 0.05, **P < 0.01, as determined by 1-way ANOVA followed by Holm-Šidák multiple-comparison test.

**Figure 6:**
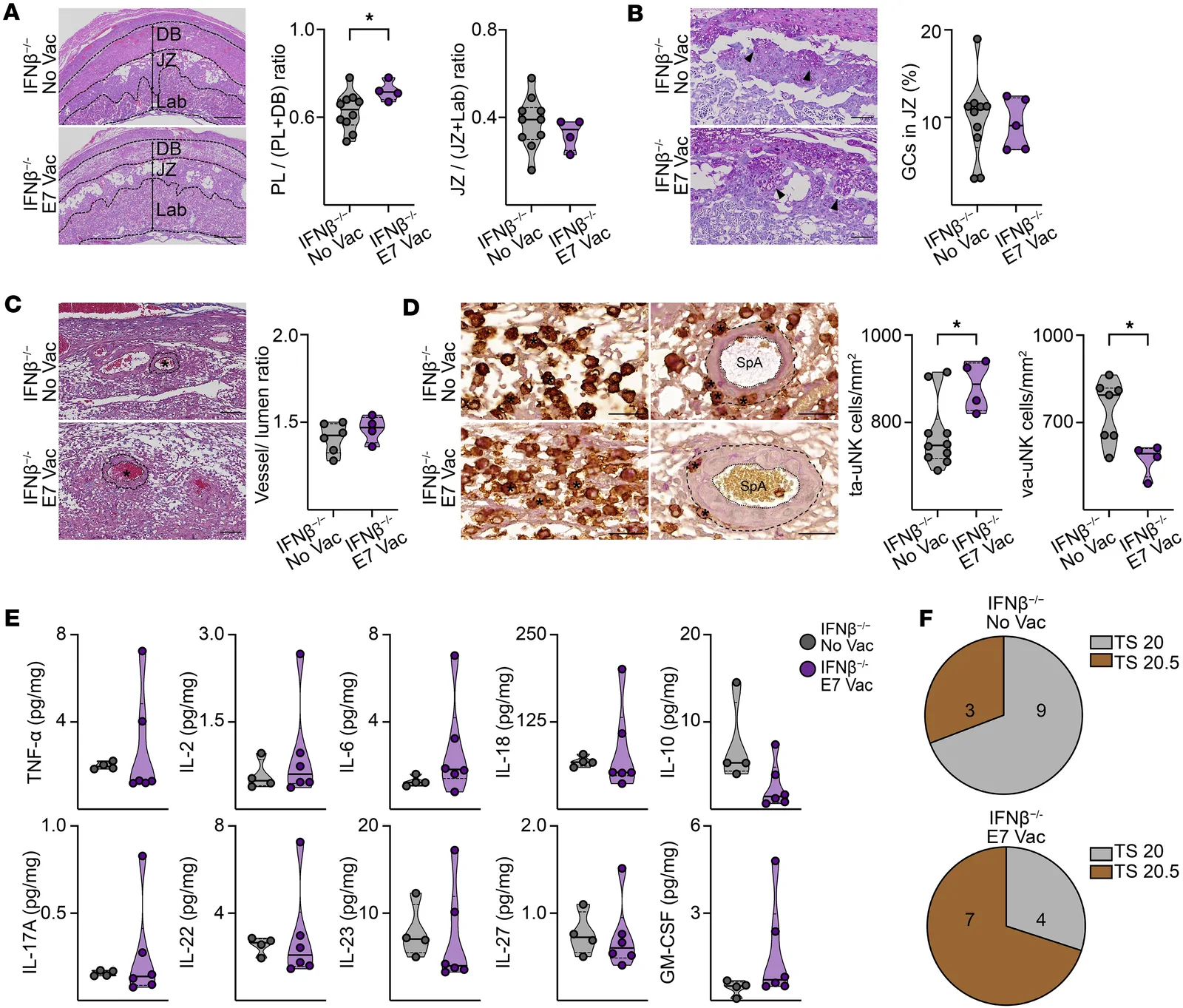
VSV-EBOV vaccination does not increase placental proinflammatory cytokine levels in IFN-β–deficient dams. Pregnant C57BL/6 *Ifnb1^–/–^* mice were challenged with 10^5^ TCID_50_ VSV-EBOV at E7 and sacrificed at 5 dpc (IFN-β^–/–^ E7 Vac). Unvaccinated IFN-β^–/–^ control dams were sacrificed at E12 (IFN-β^–/–^ No Vac). (**A**) H&E staining of E12 placental sections from unvaccinated (IFN-β^–/–^ No Vac) and vaccinated IFN-β^–/–^ dams (IFN-β^–/–^ E7 Vac). Scale bars: 500 μm. Quantification shows the relative proportion of placental labyrinth (PL) depth to combined PL and decidua basalis (DB) depth (left), and junctional zone (JZ) to combined JZ and labyrinth (Lab) depth (right). (**B**) PAS staining of E12 placental sections to assess glycogen-rich cells. Arrowheads indicate glycogen cells (GCs). Scale bars: 100 μm. The graph shows the proportion of PAS^+^ cells (GCs) within the JZ. (**C**) Masson-Goldner trichrome staining of E12 placental sections to assess SpA remodeling. Black asterisks indicate SpA. Scale bars: 100 μm. The graph shows the ratio of SpA area to luminal area. (**D**) DBA/PAS staining of E12 placental sections showing accumulation of mature uNK cells. Double-positive (DBA^+^PAS^+^) tissue-associated uNK (ta-uNK; left) and vascular-associated uNK (va-uNK; right) cells. Scale bars: 50 μm. Data in **A**–**D** are presented as mean ± SD (*n* = 4–10 per group). (**E**) Levels of TNF-α, IL-2, IL-6, IL-18, IL-10, IL-17A, IL-22, IL-23, IL-27, and GM-CSF were measured in placental lysates from unvaccinated (IFN-β^–/–^ No Vac) and vaccinated IFN-β^–/–^ dams (IFN-β^–/–^ E7 Vac) at 5 dpc using Luminex multiplex analysis. (**F**) Fetal developmental stage at E12 assessed by Theiler staging (TS), presented as pie charts. Number of fetuses per group is indicated within each segment. Data are shown as mean ± SD. *P < 0.05 vs. unvaccinated IFN-β^–/–^ controls, as determined by 2-tailed Mann-Whitney *U* test.

**Table 1:**
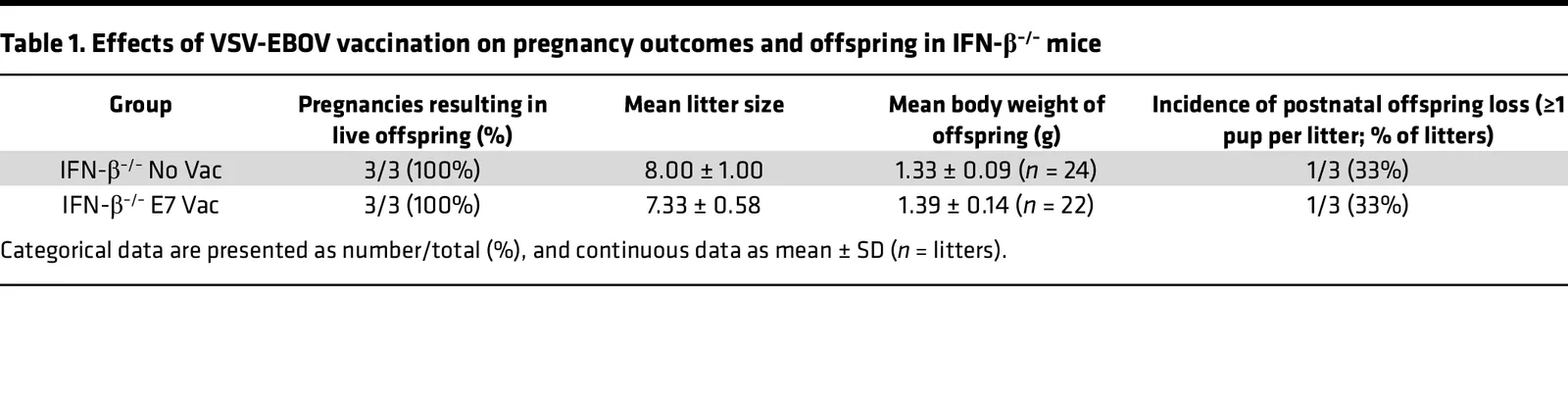
Effects of VSV-EBOV vaccination on pregnancy outcomes and offspring in IFN-β^–/–^ mice

## Version 1 10/22/2026

Electronic publication
