# Supplementary material for: Immunologic and placental responses to the VSV-EBOV vaccine in a pregnant mouse model
Source: JCI Insight. 2026 Oct 22;11(20):e201907. doi: 10.1172/jci.insight.201907 (PMC13726559; doi:10.1172/jci.insight.201907)
Supplement: Supplementary file 1 [file jciinsight-11-201907-s201.pdf]

Supplemental Information

Immunologic and Placental Responses to the VSV-EBOV Vaccine in a Pregnant Mouse Model

Aaron P. Lewkowicz, Yiru Wang, Mirian I Mendoza, Kaliroi Engel, Logan Kelley-Baker, Iara Rattner, Amber Lopez, Ian L. McWilliams, Derek D.C. Ireland, Mohanraj Manangeeswaran, Ha-Na Lee, Daniela Verthelyi, Sandra M. Blois

Supplemental Methods

RNA extraction and real-time qPCR

Placentas derived from E7 vaccinated- and PBS- treated dams on E12 were thawed in Trizol and RNA isolated (per Trizol manufacturers' protocol). RNA concentration and purity were determined by spectrophotometry at 260 nm and 280 nm using a NanoDrop 1000 spectrophotometer (ThermoFisher). For cDNA, 1 µg of RNA was reverse transcribed with random primers using Multiscript High-Capacity Reverse Transcriptase (ThermoFisher) per manufacturer's protocol after receiving DNase treatment (DNA-free Turbo kit, ThermoFisher) and stored at −20°C. Gene quantification from cDNA was performed using a Viia7 real-time PCR machine with Quant Studio software. Gene fold change in expression (ΔΔCt) was determined by normalizing Ct values to GAPDH (housekeeping gene) and then to E12.5 placentas from E7 PBS treated control dams.

Virus quantification by real-time qPCR

We used 1 µg of isolated total RNA for each sample, and VSV-N RNA levels are expressed as VSV- N copies/µg of total RNA using a standard curve, as previously described.(1) Briefly, one-step reverse transcription (RT)-real time qPCR was performed on a 10-fold serial dilution of this standard RNA in parallel with 1 µg of unknown sample RNA. This assay amplifies VSV genome position 1083 to 1144 based on wild type VSV (Indiana serotype). Reaction parameters for the Applied Biosystems Viia7 real time PCR machine: 60°C for 30 min for reverse transcription, followed by 95°C for 15 min to inactivate the RT enzyme and activate the DNA polymerase followed by 45 cycles of 95°C, 15 sec and 60°C for 1 min.

Gene expression analysis

mRNA expression levels of immune-related genes were determined using the Nanostring nCounter gene expression system (NanoString Technologies, Seattle, WA). Isolated RNA (100 ng) was hybridized with probes from nCounter Mouse Inflammation v2 Panel at 65 °C for 18 h. Hybridized products were prepared for cartridge loading on an nCounter PrepStation. Digital Counting of fluorescent signals was conducted using the nCounter Digital Analyzer. Data analysis including statistics was carried out with the nSolver 4.0 software.

Supplemental Figures

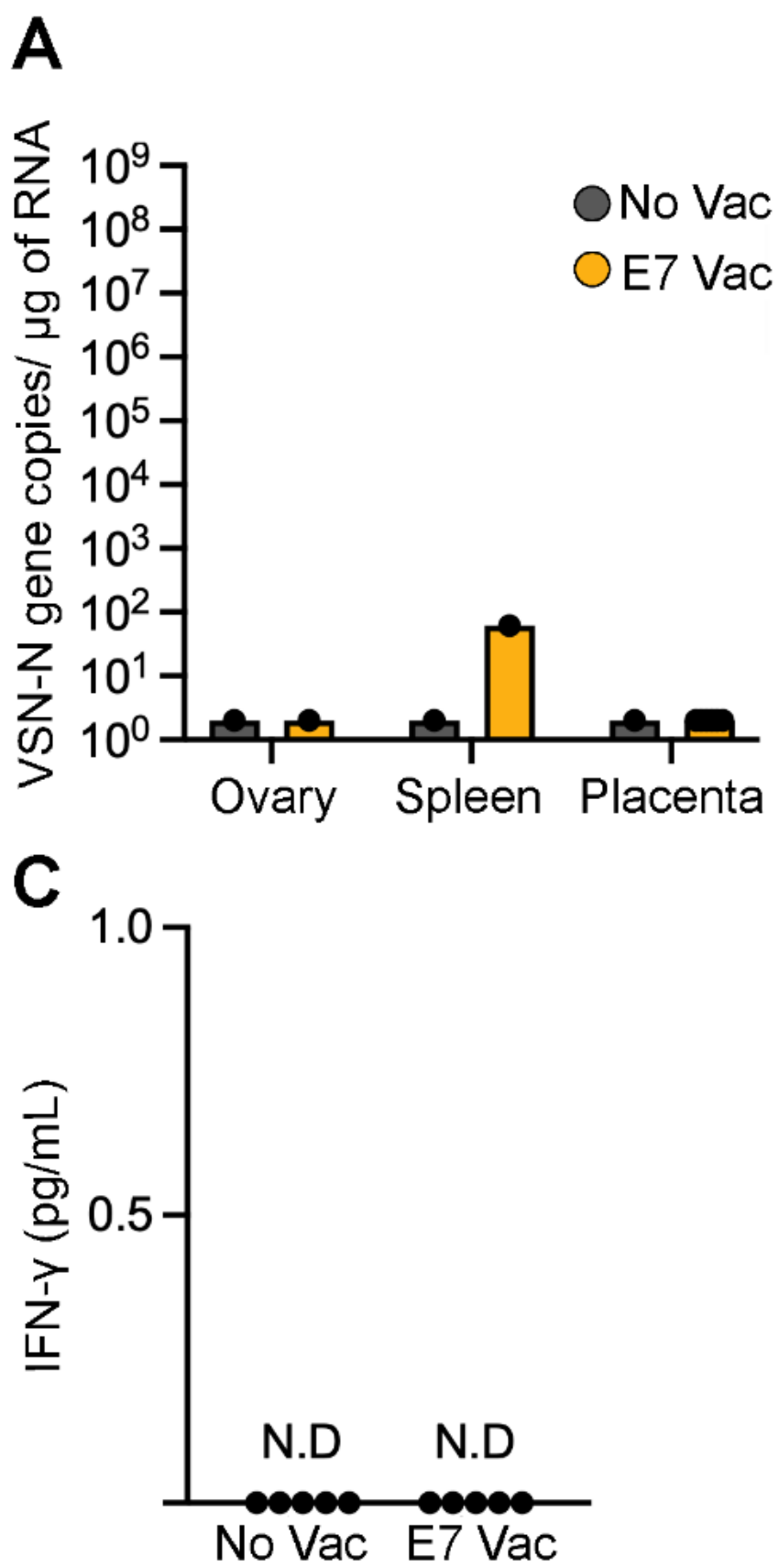

Figure S1. Absence of detectable viral RNA in maternal and placental tissues with modest induction of proinflammatory gene expression following VSV-EBOV vaccination at 2 days post-challenge (dpc). Pregnant C57BL/6 mice were challenged with 1×10^5 $TCID_{50}$ of VSV-EBOV at E7 and sacrificed at 2 dpc. (**A**) RNA isolated from ovary, spleen, and placenta of unvaccinated and VSV-EBOV–vaccinated dams was analyzed for VSV nucleoprotein (VSV-N) expression. (**B**) Relative mRNA expression of *Ifna2, Ifnb1, Ifng, Cxcl10, Il6, Tnf,* and *Klrc1* in placental tissue. (**C**) Serum IFN-γ protein levels in control and VSV-EBOV–vaccinated dams at 5 dpc, measured by Luminex assay. Data are presented as mean ± SD (B: n = 4-6 per group; C: n = 5 per group).

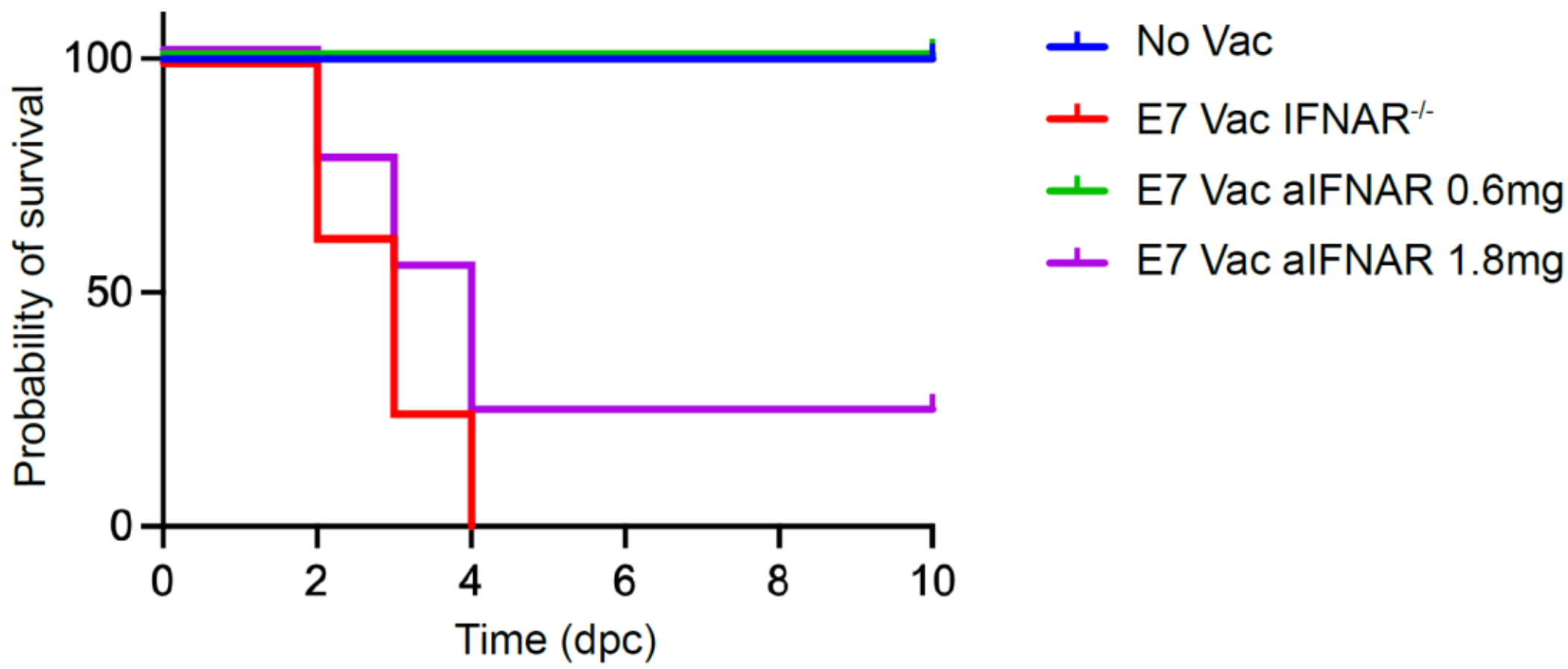

Figure S2. Complete IFNAR blockade results in maternal mortality following VSV-EBOV vaccination. Pregnant C57BL/6 mice were administered anti-IFNAR blocking antibodies (0.6 or 1.8 mg, i.p.) at E6 and subsequently challenged with 1×10^5 $TCID_{50}$ of VSV-EBOV at E7. Control groups included untreated pregnant C57BL/6 wild type mice and pregnant IFNAR-deficient mice (*Ifnar1*-/-), both challenged with 1×10^5 $TCID_{50}$ of VSV-EBOV at E7. Mice were monitored longitudinally for survival.

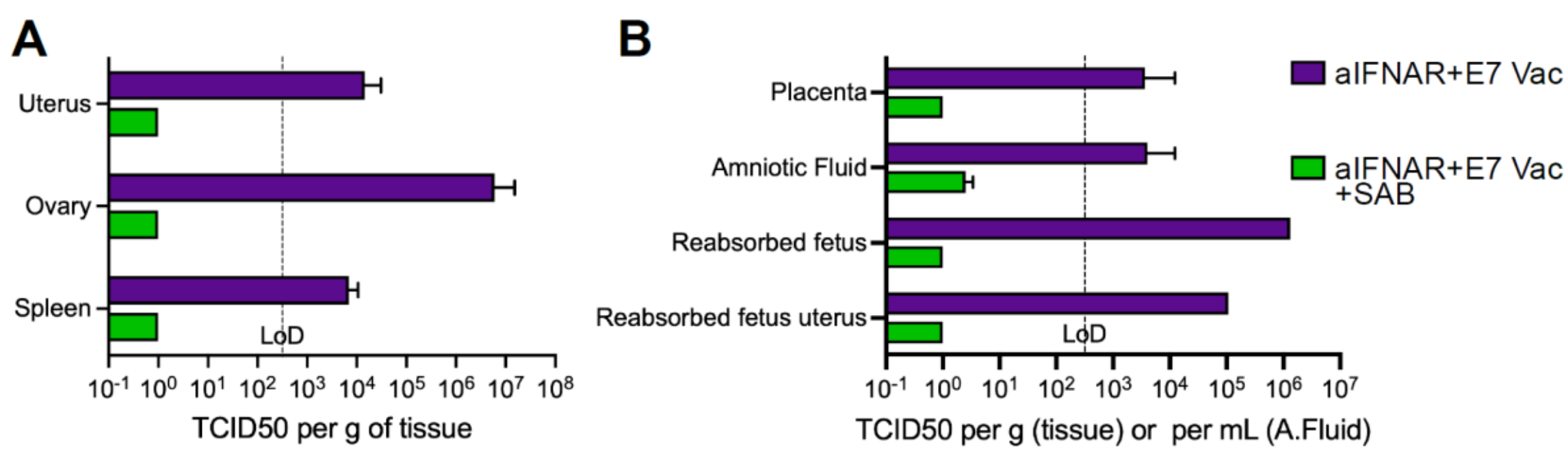

Figure S3. EBOV glycoprotein mediates VSV-EBOV infection in IFNAR-blocked dams and embryos. Pregnant C57BL/6 mice were treated at E6 with anti-IFNAR–blocking antibodies (600 µg, i.p.) in the presence or absence of anti-EBOV glycoprotein antibodies (SAB-139; 2.5 mg, i.p.), followed by challenge with 1×10^5 $TCID_{50}$ of VSV-EBOV at E7. Dams were sacrificed at E12. (**A**-**B**) Viral titers in maternal tissues (**A**) and embryos (**B**) were quantified by $TCID_{50}$ assay. The dashed line indicates the limit of detection (LoD = 316 $TCID_{50}$).

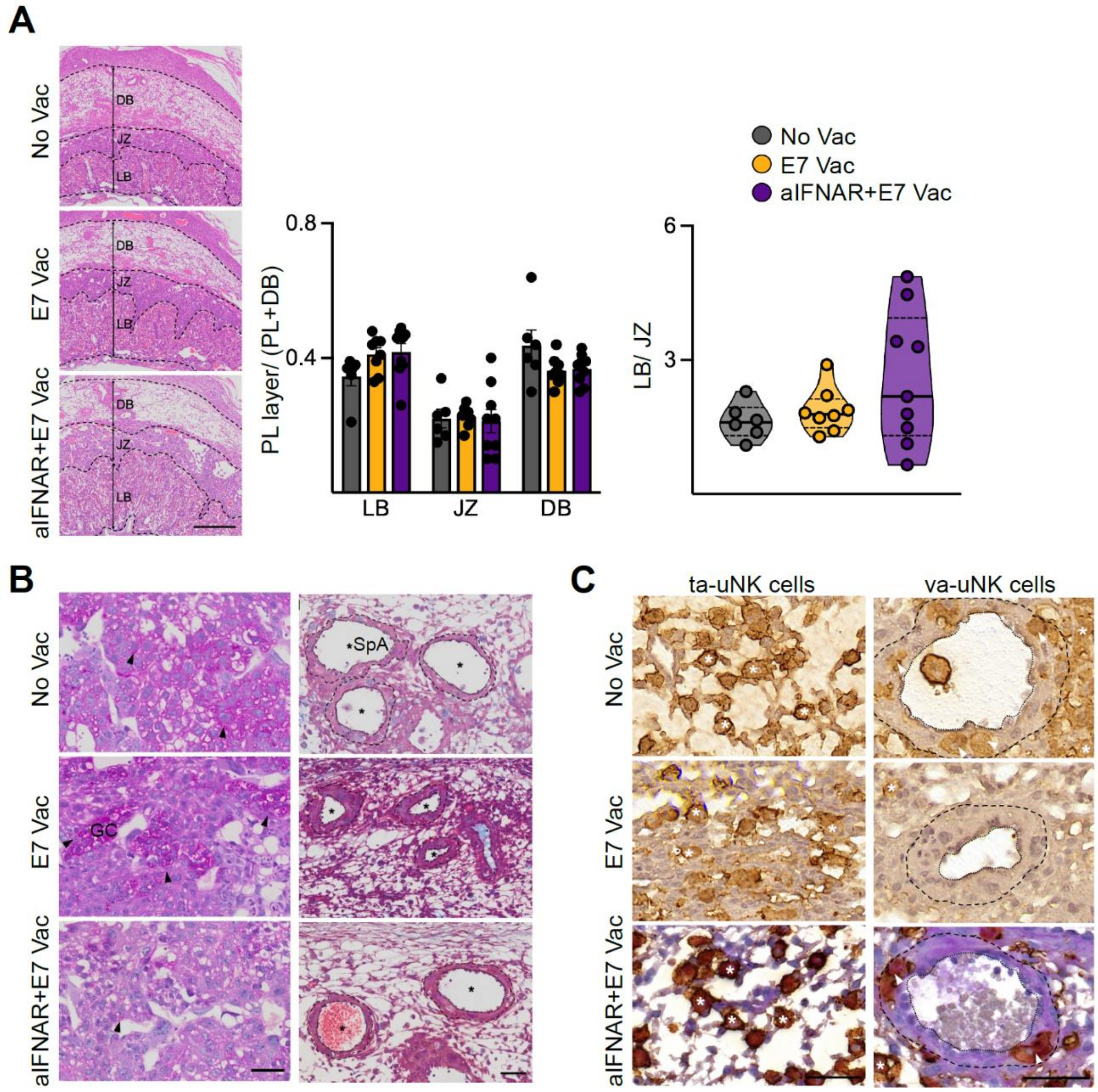

Figure S4. Blockade of type I IFN signaling prevents placental alterations in VSV-EBOV-vaccinated dams. Pregnant C57BL/6 mice were treated with or without anti-IFNAR–blocking antibodies (0.6 mg, i.p.) at E6 and challenged with $1{\times}10^5$ $TCID_{50}$ of VSV-EBOV at E7, followed by sacrifice at E12. Uninfected pregnant C57BL/6 mice served as controls and were also sacrificed at E12. (**A**) Representative H&E-stained sections of E12 placentas from unvaccinated and VSV-EBOV–vaccinated dams with or without anti-IFNAR treatment. Quantification shows the relative proportions of labyrinth (LB), junctional zone (JZ), and decidua basalis (DB) areas (left), and the ratio of LB to JZ (right). Scale bar = 500 µm. (**B**) Representative images of placental glycogen cells (left) and maternal spiral artery (SpA) wall structure (right) from the indicated groups. Glycogen cells were visualized by PAS staining, and SpA morphology was assessed by Masson-Goldner staining. Scale bar = 50 µm. (**C**) DBA (Dolichos biflorus agglutinin)-PAS (Periodic acid–Schiff) staining of E12 placental sections showing accumulation of mature uterine natural killer (uNK) cells. Double-positive (PAS^+DBA^+) tissue-associated uNK (ta-uNK; left) and vascular-associated uNK (va-uNK; right) cells are indicated by white asterisks. Scale bar = 50 µm.

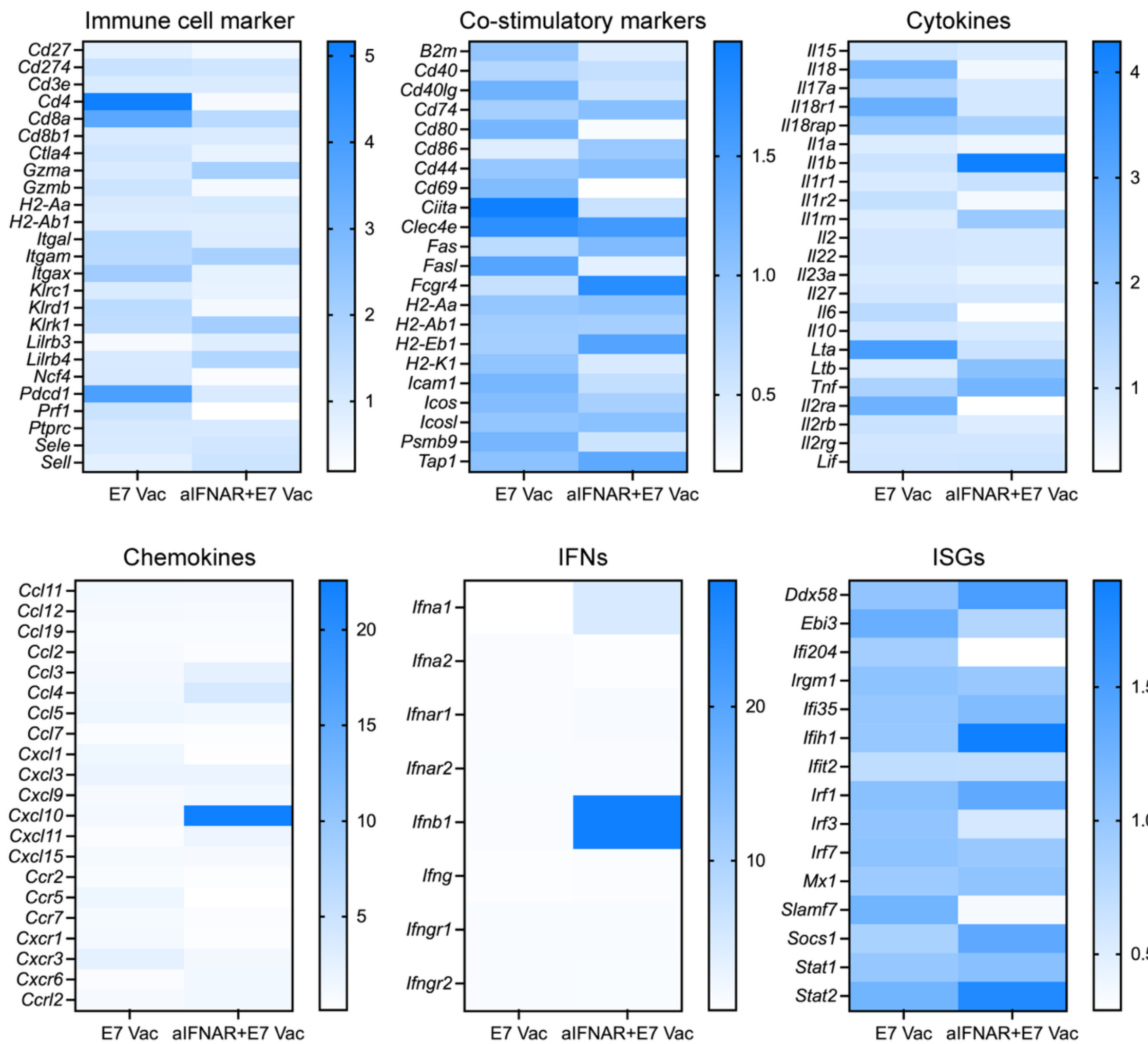

Figure S5. Placental gene expression following VSV-EBOV infection with or without IFNAR blockade. Pregnant C57BL/6 mice received anti-IFNAR antibodies (0.6 mg, i.p.) at embryonic day (E) 6 and were challenged with 10^5 $TCID_{50}$ VSV-EBOV at E7. Animals were sacrificed at E12. Uninfected pregnant C57BL/6 mice served as controls (No Vac) and were also collected at E12. Heatmap showing mRNA expression of selected genes from a 591-gene panel in placental tissue at 5 days post–viral challenge (dpc) from infected dams with or without anti-IFNAR pretreatment (n= 3-4 per group). Gene expression was quantified using the NanoString nCounter Mouse Immunology Panel and normalized to the mean expression of the uninfected control group (n = 4).

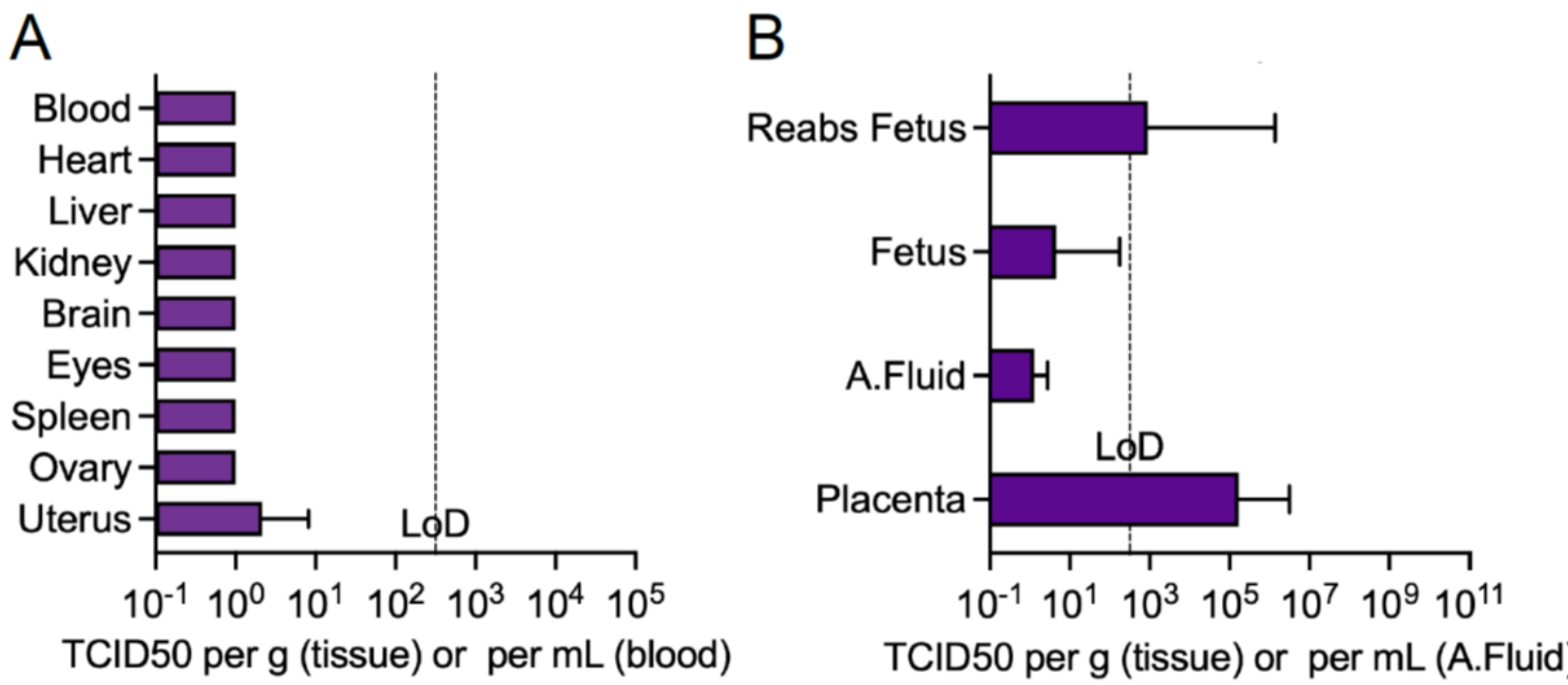

Figure S6. Reduced viral load in vaccinated mice despite blockade of type I IFN signaling at 11 days post-challenge. Viral titers in maternal (**A**) and embryonic (**B**) tissues were quantified by $TCID_{50}$ assay at 11 days post-challenge. The dashed line indicates the limit of detection (LoD = 316 $TCID_{50}$).

Table S1. Effects of VSV-EBOV vaccination on pregnancy outcomes and offspring

Group	Pregnancies resulting in live offspring (%)	Mean litter size	Mean body weight of offspring (g)	Incidence of postnatal offspring loss (≥1 pup per litter, % of litters)
No Vac	10/10 (100%)	7.5 ± 1.73 (n=5)	1.42 ± 0.13 (n=38)	1/10 (10%)
E7 Vac	**6/8 (75%)**	6.67 ± 2.50 (n=6)	**1.25 ± 0.12 (n=32)** ****	**3/6 (50%)**
E10 Vac	5/5 (100%)	7.4 ± 2.3 (n=5)	**1.22 ± 0.11 (n=37)** ****	**4/5 (80%)**
E15 Vac	6/6 (100%)	5.5 ± 1.9 (n=6)	**1.30 ± 0.12 (n=33)** ***	1/6 (16%)

Categorical data are presented as number/total (%), and continuous data as mean ± SD, with *n* indicating the number of litters or offspring as specified. Statistical analyses were performed using 1-way ANOVA followed by Dunnett's multiple-comparison test for continuous variables and Fisher's exact test for categorical variables. ***$P < 0.001$, ****$P < 0.0001$vs. No Vac control.

References

1. Lee HN, et al. Ebola virus-induced eye sequelae: a murine model for evaluating glycoprotein-targeting therapeutics. *EBioMedicine.* 2024;104:105170.
